# Nicotinic acetylcholine receptors (nAChRs) are expressed in Trpm5 positive taste receptor cells (TRCs)

**DOI:** 10.1371/journal.pone.0190465

**Published:** 2018-01-02

**Authors:** Jie Qian, Shobha Mummalaneni, John R. Grider, M. Imad Damaj, Vijay Lyall

**Affiliations:** 1 Physiology and Biophysics Virginia Commonwealth University, Richmond, VA, United States of America; 2 Pharmacology and Toxicology, Virginia Commonwealth University, Richmond, VA, United States of America; The University of Tokyo, JAPAN

## Abstract

Nicotine evokes chorda tympani (CT) taste nerve responses and an aversive behavior in Trpm5 knockout (KO) mice. The agonists and antagonists of nicotinic acetylcholine receptors (nAChRs) modulate neural and behavioral responses to nicotine in wildtype (WT) mice, Trpm5 KO mice and rats. This indicates that nicotine evokes bitter taste by activating a Trpm5-dependent pathway and a Trpm5-independent but nAChR-dependent pathway. Rat CT responses to ethanol are also partially inhibited by nAChR blockers, mecamylamine and dihydro-β-erythroidine. This indicates that a component of the bitter taste of ethanol is also nAChR-dependent. However, at present the expression and localization of nAChR subunits has not been investigated in detail in taste receptor cells (TRCs). To this end, *in situ* hybridization, immunohistochemistry and q-RT-PCR techniques were utilized to localize nAChR subunits in fungiform and circumvallate TRCs in WT mice, Trpm5-GFP transgenic mice, nAChR KO mice, and rats. The expression of mRNAs for α7, β2 and β4 nAChR subunits was observed in a subset of rat and WT mouse circumvallate and fungiform TRCs. Specific α3, α4, α7, β2, and β4 antibodies localized to a subset of WT mouse circumvallate and fungiform TRCs. In Trpm5-GFP mice α3, α4, α7, and β4 antibody binding was observed in a subset of Trpm5-positive circumvallate TRCs. Giving nicotine (100 μg/ml) in drinking water to WT mice for 3 weeks differentially increased the expression of α3, α4, α5, α6, α7, β2 and β4 mRNAs in circumvallate TRCs to varying degrees. Giving ethanol (5%) in drinking water to WT mice induced an increase in the expression of α5 and β4 mRNAs in circumvallate TRCs with a significant decrease in the expression of α3, α6 and β2 mRNAs. We conclude that nAChR subunits are expressed in Trpm5-positive TRCs and their expression levels are differentially altered by chronic oral exposure to nicotine and ethanol.

## Introduction

Nicotine (Nic) is bitter tasting. In some inbred strains of rats and mice, ethanol (ETOH) has both a sweet taste quality and a bitter taste quality [1]. In a previous study [2], we compared behavioral and chorda tympani (CT) taste nerve responses to Nic and quinine in wildtype (WT) mice and Trpm5 knockout (KO) mice. Trpm5 KO mice were indifferent to quinine. However, Trpm5 KO mice responded to Nic with a dose-dependent increase in the CT response and in behavioral experiments found Nic to be aversive. In addition, we observed that both in WT mice and in Trpm5 KO mice mecamylamine (Mec), a broad spectrum blocker of nAChRs, decreased the magnitude of the CT response to Nic and its aversiveness [2]. These studies led us to conclude that unlike quinine, Nic can transduce bitter taste by interacting with two independent but parallel receptor-mediated pathways. One pathway is shared by Nic, quinine and other bitter compounds, and is dependent upon the presence of G-protein coupled bitter taste receptors (GPCRs, T2Rs) and Trpm5 expressed in bitter sensing taste receptor cells (TRCs) [2]. The second pathway is independent of Trpm5, and involves the interaction of Nic with nAChRs expressed in a subset of TRCs. In a follow up study [3], we further demonstrated that, in addition to Nic, CT responses to acetylcholine and ETOH are also blocked by Mec, dihydro-β-erythroidine (DHβE), and CP-601932 (a partial agonist of α3β4* nAChR; *denotes that other subunits may be part of the nAChR complex) [4]. We have also demonstrated that protein kinase A (PKA) and protein kinase C (PKC) modulators that regulate the activity of nAChRs [5–7] also regulate CT responses to Nic and ETOH [3]. Taken together, the above results indicate that CT responses to Nic and ETOH that are not dependent upon Trpm5 are due to the interactions of Nic and ETOH with nAChRs expressed in a subset of fungiform (FF) TRCs [3].

The expression of nAChRs has been demonstrated in STC-1 cells [8] that have been shown to express functional taste receptors involved in the transduction of all five primary taste qualities [9]. In addition, nAChRs have been shown to be expressed in small intestinal enteroendocrine cells [8], epithelial cells of intrapulmonary airways that also co-express bitter taste transduction signaling molecules [10], in bronchial epithelial cells [11], in colonic mucosal epithelium [12], in epithelial cells of the small bowel [13], and human proximal tubular epithelial (HK-2) cells [14].

In addition to the classical sweet, bitter, umami, salty and sour taste receptors, nAChRs expressed in central and peripheral organs also play a major role in the regulation of appetite and body weight [15], and in the neurobiological effects of ETOH [16]. In line with these observation, alterations in CT responses to Nic and ETOH by nAChR agonists and antagonists suggest that DHβE-sensitive nAChRs composed of α4β2 and CP-601932-sensitive nAChRs composed of α3β4* subunits are most likely involved in Nic- and ETOH-induced increase in the CT response. Activation of nAChR results in membrane depolarization [17]. However, at present, the information regarding the identity of the functional nAChRs and the specific taste cell type in which they are expressed remains to be elucidated. Also, it is not known if nAChR expression can be altered in TRCs by acute or chronic oral Nic and ETOH exposure. Accordingly, here, we used *in situ* hybridization (ISH), immunohistochemistry (IHC) and qRT-PCR techniques to localize nAChR subunits in fungiform (FF) TRCs, circumvallate (CV) TRCs, and intestinal enteroendocrine cells in wildtype (WT) mice, Trpm5-GFP transgenic mice, nAChR knockout (KO) mice, and rats. Our results indicate that nAChR subunits are expresses in Trpm5-positive TRCs and intestinal enteroendocrine cells. Our results also demonstrate that nAChR mRNA expression levels are differentially altered by chronic oral exposure to nicotine and ethanol.

## Materials and methods

### Animals

Sprague-Dawley (SD) rats (150–200 g) and wildtype (WT; C57BL/6J, 30-40g) mice were obtained from Charles Rivers Laboratories, Wilmington, MA, USA. Trpm5-GFP transgenic mice were originally generated in Robert F. Margolskee’s laboratory at the Monell Chemical Senses Center, Philadelphia, PA [18]. The transgeneic mice were bred at VCU animal facility from breeding pairs obtained from Dr. Margolskee. Mice null (KO) for the nAChR subunit α4 (provided by Dr. Henry Lester at the California Institute of Technology, with the permission of Dr. John Drago) [19], nAChR α7 subunit KO mice (obtained from Jackson laboratories, Bar Harbor, ME), and their wild-type littermates were bred in an animal care facility at Virginia Commonwealth University. Mice were housed in a 21°C humidity-controlled Association for Assessment and Accreditation of Laboratory Animal Care (AALAC)-approved animal care facility. All animals were maintained on 12h light/dark schedule and were given *ad libitum* pellet diet and water. All *in vivo* animal protocols were approved by the Institutional Animal Care and Use Committee (IACUC) of VCU (Protocol #AD20116).

### *In situ* hybridization (ISH)

#### RNA probes

The cDNA fragments of nAChR subunits α4, α7, β2, and β4 were obtained by RT-PCR from circumvallates (CV) and fungiform (FF) taste tissue of C57BL/6 mice or SD rats. The cDNA fragments were cloned into pSPT19 vector between EcoR I and Hind III restriction site. Plasmid DNAs were purified by using QIAprep Spin Miniprep Kit (Qiagen, Cat 27104) and sequenced at VCU DNA core lab. Template DNAs were digested with Nde I and transcribed by T7 RNA polymerase for antisense (AS) probes, or they were digested with Nhe I and transcribed by Sp6 RNA polymerase for sense (S) probes. Probes were generated with the DIG RNA Labeling kit (Roche Diagnostics USA, Cat number 11175025910) or Fluorescein RNA labeling Mix (Roche Diagnostics USA, Cat. Number 11685619910). Dot-blot assay were used to determine the labeling efficiency. Primers used for *in situ* hybridization probes are shown in Table 1.

**Table 1:** Primers used to generate *in situ* hybridization probes.

Species	Gene	Primer	NCBI Reference Number
Mouse	nAChR a4 F	CCGGAATTCCTCGTCTAGAGCCCGTTCTG	NM_015730.5
	nAChR a4 R	CCGAAGCTTGTCCGCGTTGTTGTAGAGGA	
Mouse	nAChR a7 F	CCGGAATTCTCATTCTTCTGAATTGGTGTGC	NM_007390.3
	nAChR a7 R	CCGAAGCTTTCTCGTCCTCCAGATTCTCTTC	
Mouse	nAChR b2 F	CCGGAATTCGGTGTTCCTGCTGCTCATCTC	NM_009602.4
	nAChR b2 R	CCGAAGCTTCTCACACTCTGGTCATCATCT	
Mouse	nAChR b4 F	CCGGAATTCCTCTCTGTTCGCTCTGCTTCA	NM_148944.4
	nAChR b2 R	CCGAAGCTTAACACGATGTCAGGCAACCA	
Rat	Trpm5 F	CCGGAATTCCAACAAGCCTGACTTCGTGC	NM_001191896.1
	Trpm5 R	CCGAAGCTTCCAAAGGAACAGGTCCCTCC	
Rat	nAChR a7 F	CCGGAATTCTCTCCTCTATAACAGTGCTGA	NM_012832.3
	nAChR a7 R	CCGAAGCTTTGCAGGCAGCAAGAATACCA	

#### Tissue preparation

The animals were anesthetized using isoflurane in a glass desiccator jar in a certified ducted chemical hood before transcardial perfusion with 4% paraformaldehyde. Mice were placed in the glass desiccator jar with a lid containing a wet cotton ball with a mixture of 20% isoflurane (v/v) in propylene glycol for 1 min. In the case of rats, a mixture of 30% isoflurane (v/v) in propylene glycol was used and the rats were kept in the closed glass desiccator jar for 2–3 min. Once the animals remained in the deep anesthetic stage for 30s they were perfused with 4% paraformaldehyde/1 × PBS for 5–10 min [20]. The tongue and brain were excised and fixed in 4% paraformaldehyde/1 × PBS for another 2 h at 4°C, and dehydrated in 40% sucrose/1 × PBS overnight at 4°C before embedding in O.C.T. Compound (Andwin Scientific, Cat 14-373-65). Sections (10 μm thick) were prepared using a CM3050S cryostat (Leica Microsystems) and applied on pre-coated microscope slides (Fisher Scientific, Cat 12-550-15). The sections were dried at room temperature for 20 min and immediately used for *in situ* hybridization or stored at −80°C for immunohistochemistry.

ISH was performed as described by Yee et al. [21]. In brief, freshly prepared sections were fixed with 4% paraformaldehyde/1 × PBS for 10 min at 4°C, permeabilized with 10 μg/ml proteinase K (Roche Applied Science, Cat 03115836001), post fixed with 4% paraformaldehyde/1 × PBS for 10 min at 4°C, and then acetylated for 10 min. All steps were followed by three 5-min washes with 1 × PBS. All solutions were prepared with diethylpyrocarbonate-treated double distilled H_2_O. Slides were prehybridized with hybridization buffer for 1 h at room temperature, the sections were hybridized with 200 ng/ml antisense (AS) riboprobes for 24 h at 56°C. Sense (S) riboprobes were used as negative control. After hybridization, the sections were washed in saline/sodium citrate (SSC) buffer and blocked with 0.3% H_2_O_2_ and blocking reagent (PerkinElmer) in Tris-buffered saline. The fluorescence signal was amplified by using TSA PLUS Fluorescence Kits (PerkinElmer cat NEL745001KT). In addition, ISH experiments were done on brain tissue as positive control to confirm the quality and specificity of the RNA probes. The hybridization buffer contained: 50% (vol/vol) deionized formamide, 5 × SSC, 5 × Denhardt’ s solution, 500 μg/mL salmon sperm DNA, 250 μL/mL of yeast tRNA, and 2.5 M EDTA in DEPC-treated water.

### Immunohistochemical (IHC) localization of nAChRs

Mice were placed in the glass desiccator jar with a lid containing a wet cotton ball with a mixture of 20% isoflurane (v/v) in propylene glycol for 1 min. Once the mice remained in the deep anesthetic stage for 30s they were transcardially perfused with 0.1 M phosphate-buffered saline (PBS) followed by 4% para-formaldehyde (PFA) in 0.1 M PBS. Excised tongues were fixed in 4% PFA for 2 h and dehydrated in 30% sucrose overnight at 4°C. Blocks were made by embedding in OCT compound. Eight μm thick cryostat sections were made at -20°C and thaw mounted on Superfrost Microscope slides. Slides were washed with PBS and processed for detection of nAChRs following the suggested protocol for using Tyramide Signal Amplification (TSA) Fluorescence System for Immunohistochemistry (Perkin-Elmer). In brief, the endogenous peroxide activity was quenched with 0.1 M PBS containing 0.3% H_2_O_2_ for 30 min followed by blocking nonspecific binding with the TNB blocking buffer (provided in the kit) and normal donkey serum for 1 h. Primary antibody was diluted in TNB buffer and the slides were incubated for 18 to 48 h at 4°C. The sections were washed with 0.3% Triton-X in 0.1 M PBS (PBST) and incubated with secondary antibody for 60–90 minutes followed by streptavidin conjugated horseradish peroxidase for 30 min. The slides were washed with PBST and incubated with Biotinyl Tyramide Amplification Reagent for 10 min. Following PBST washing, the slides were incubated in a Streptavidin Fluorophore conjugate for 10 minutes. The slides were then washed and coverslipped using Fluoroshield with 4,6-diamidino-2-phenylindole (DAPI) mounting media (Sigma). Slides were viewed with LSM 700 Laser Scanning Microscope and confocal pictures were taken using ZEN 2011 software. In some cases, slides were viewed using a Nikon ECLIPSE E800M microscope equipped with UV, FITC, TRITC, Cy5, GFP and FITC+TRITC filter sets, and a Diagnostic Instruments Spot RT CCD camera.

#### Antibodies

The primary AChR α3 Rabbit polyclonal antibody (SC-5590; 1:50–1:500), AChR α4 Rabbit polyclonal antibody (SC-5591; 1:50–1:500), AChR α7 Goat polyclonal antibody (SC-1447; 1:50–1:200), and AChR β2 Rabbit polyclonal antibody (SC-11372; 1:50–1:500) were obtained from Santa Cruz Biotecnology. The AChR β4 Goat polyclonal antibody (ab-129276; 1:50–1:500) was obtained from AbCam Inc. The secondary antibodies Donkey anti Goat (SC-2042; 1:500) and Donkey anti Rabbit (SC-2089; 1:500) were obtained from Santa Cruz Biotecnology. The Fluorophore used was SA-Fluorescein. In Trpm5-GFP transgenic mice the α3 and β4 nAChR subunits were detected using secondary donkey anti rabbit antibody (SC-2089) or donkey anti goat (SC-2042), respectively, and a Cyamine 5 conjugated amplification reagent. In the intestinal crypt cells from the Trpm5-GFP transgenic mice, α3 antibody binding was detected using a non TSA method (without amplification) using a Donkey anti Rabbit 590-secondary antibody A-11012 (Life Technologies).

### Chronic nicotine (Nic) and ethanol (ETOH) exposure

WT mice were given (-) nicotine free base (Sigma) and ETOH (Sigma) in drinking water. Chronic oral administration of Nic (50–200 μg/ml) has been shown to induce dependence and tolerance in C57BL/6 mice [22, 23]. WT mice were maintained on one bottle containing H_2_O, Nic (100 μg/ml) or ETOH (5%). Each group contained 6–8 WT mice. After 3 weeks the mice were sacrificed and their CV taste tissue was collected as described earlier [2]. The CV tissue from 6–8 mice in each group were pooled for qRT-PCR studies as described below.

### Quantitative real-time PCR (qRT-PCR)

QRT-PCR was used to measure RNA transcripts of nAChR subunits. Total RNA was purified by using the TRIzol reagent (cat# 15596018, Thermo Fisher Scientific, MA, USA) and reverse transcripted using High-Capacity cDNA Reverse Transcription Kit (Thermo Fisher Scientific, MA, USA; Cat# 4368814). Real-time PCR was conducted using carboxyfluorescein (FAM)-labeled probe sets from Integrated DNA Technologies (Coralville, IA): GAPDH: Mm.PT.39.1; Chrna3: Mm.PT.56a.43533732; chrnb4: Mm.PT.56a.11867247; Chrna4: Mm00516561_m1; Chrna5: Mm00616329_m1; Chrna6: Mm00517529_m1; Chrnb2:Mm00515323_m1. Results were calculated using the 2−ΔΔCt method based on GAPDH amplification, and normalized to the control group. The statistical analysis was performed using GraphPad ‘t’ test.

## Results

### ISH studies in mouse CV and FF TRCs

The expression of α and β nAChR subunit mRNAs was investigated in CV and FF taste papillae sections of mouse tongue using ISH technique. The mouse brain tissue was used as a positive control. The anti-sense (AS) Chrnb2, Chrna4, and Chrna7 riboprobes in mouse brain sections revealed neurons that express mRNAs for β2 nAChR (S1 Fig; P1), α4 nAChR (S1 Fig; P2), and α7 nAChR (S1 Fig; P3). Neurons that expressed α4 nAChR mRNA also expressed the β2 nAChR mRNA (S1 Fig; P2), and neurons that expressed α7 nAChR mRNA also expressed the β2 nAChR mRNA (S1 Fig; P3). These results demonstrate the validity of our ISH technique for detecting the presence of nAChRs in brain slices and taste tissue.

**Fig 1:**
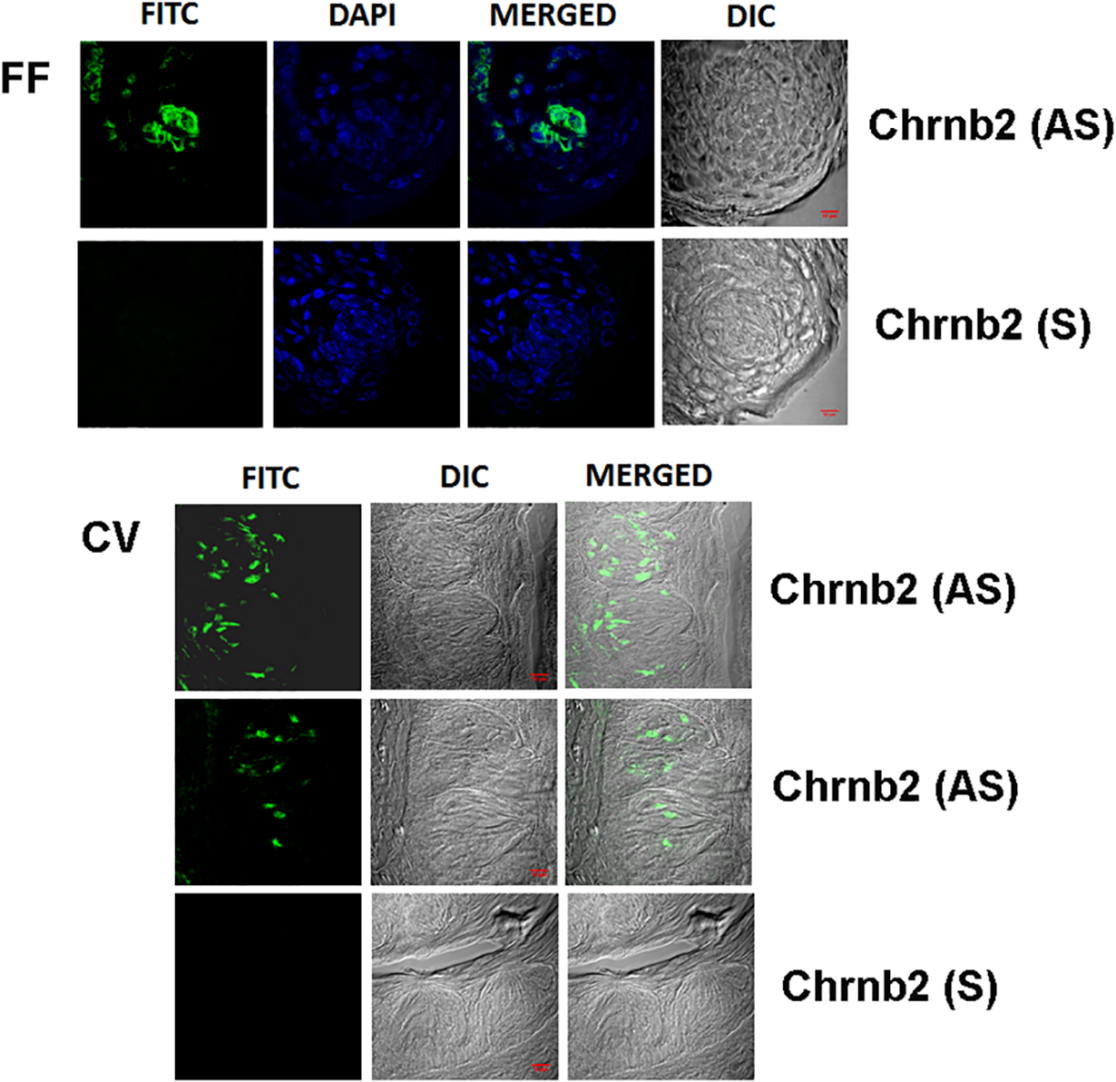
*In situ* hybridization of Chrnb2 digoxigenin labeled anti-sense RNA probe in FF and CV taste bud cells. **(FF)** Shows images of a WT mouse FF taste papilla section labelled with Alexa Fluor® 488 (FITC), DAPI, the merged image of DAPI and FITC, and the DIC image. The anti-sense (AS) Chrnb2 riboprobe labeled a subset of FF taste bud cells. No significant fluorescence was observed when the sense probe (S) was used. **(CV)** Shows low magnification images of a WT mouse CV taste papilla section labelled with Alexa Fluor® 488 (FITC), DIC image, and the merged image of FITC and the DIC image. The anti-sense (AS) Chrnb2 riboprobe labeled a subset of WT mouse CV taste bud cells. No significant fluorescence was observed when the sense (S) probe was used. Bar = 10 μm.

**Fig 2:**
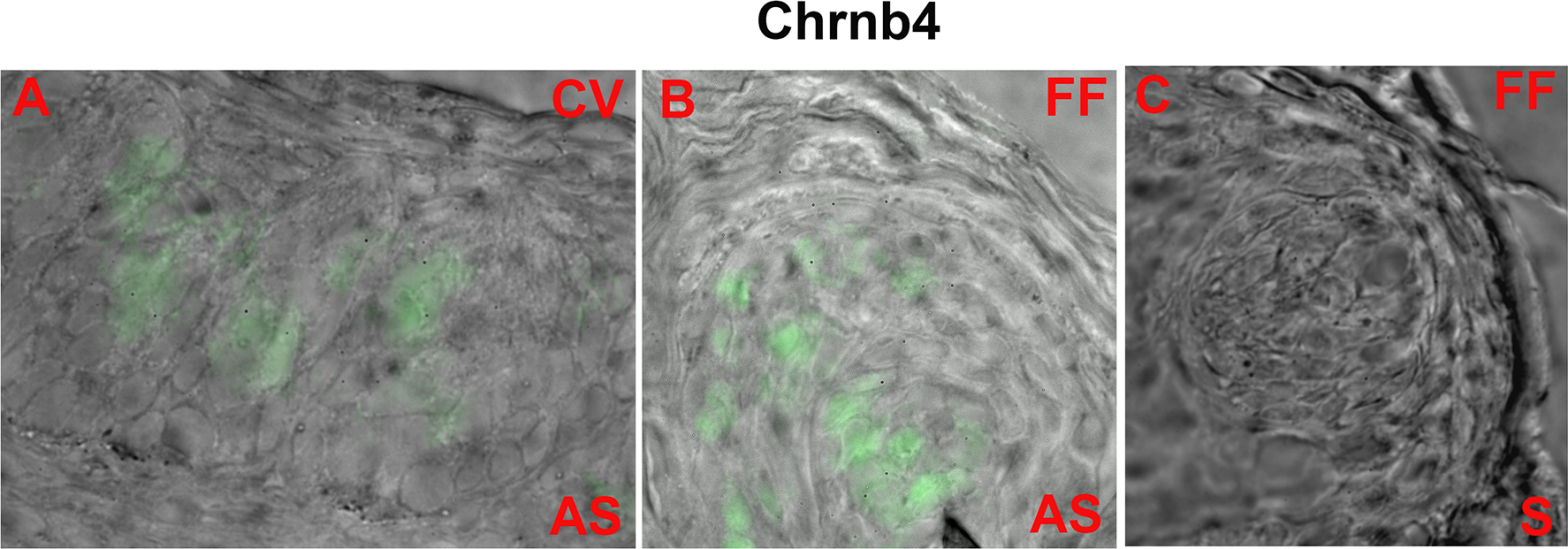
*In situ* hybridization of Chrnb4 digoxigenin labeled anti-sense RNA probe in WT mouse CV taste bud cells. Shows merged images of a WT mouse FF taste papilla section labelled with Alexa Fluor® 488 (FITC) and the DIC image. The anti-sense (AS) riboprobe Chrnb4 labeled a subset of CV taste bud cells **(A)** and FF taste bud cells **(B)**. No significant fluorescence was observed when the sense probe (S) was used in a FF taste papilla section **(C)**.

The α4, α7 and β2 nAChR mRNA expression levels in brain are high and, therefore, no amplification of the signal was needed for detecting them individually and for observing co-localization with other nAChR subunits. In contrast, due to the low expression of nAChRs in the taste tissue, we were not able to detect the ISH signal without amplification in FF and CV TRCs. Thus, all ISH studies in the taste tissues were performed with signal amplification. In a representative WT mouse FF taste papilla section (Fig 1; FF) the AS riboprobe Chrnb2 revealed that only a subset of TRCs express the mRNA for β2 nAChR. The sense (S) Chrnb2 riboprobe did not label any TRCs within the FF taste bud (Fig 1; FF). Similarly, in several rat CV sections (Fig 1; CV) only a subset of CV TRCs expressed mRNA for β2 nAChR. In separate rat CV (Fig 2A) and FF (Fig 2B) taste buds, the AS Chrnb4 riboprobe revealed only a subset of TRCs that express the mRNA for β4 nAChR.

**Fig 3:**
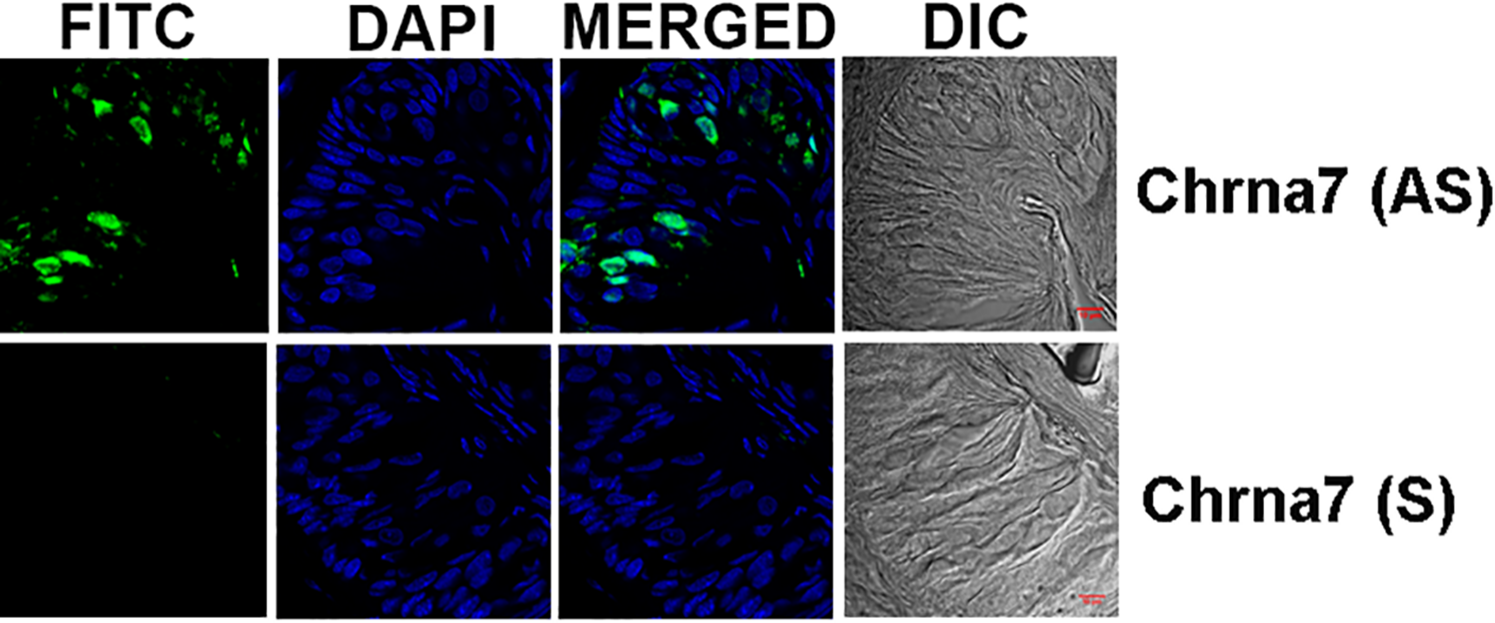
*In situ* hybridization of Chrna7 digoxigenin labeled anti-sense RNA probe in rat CV taste papillae sections. Shows images of rat CV papilla sections labelled with Alexa Fluor® 488 (FITC), DAPI, the merged images of FITC and DAPI, and the DIC image. The anti-sense (AS) Chrna7 riboprobe labeled a subset of rat CV taste bud cells (AS). No significant fluorescence was observed when the sense (S) probe was used. Bar = 10 μm.

**Fig 4:**
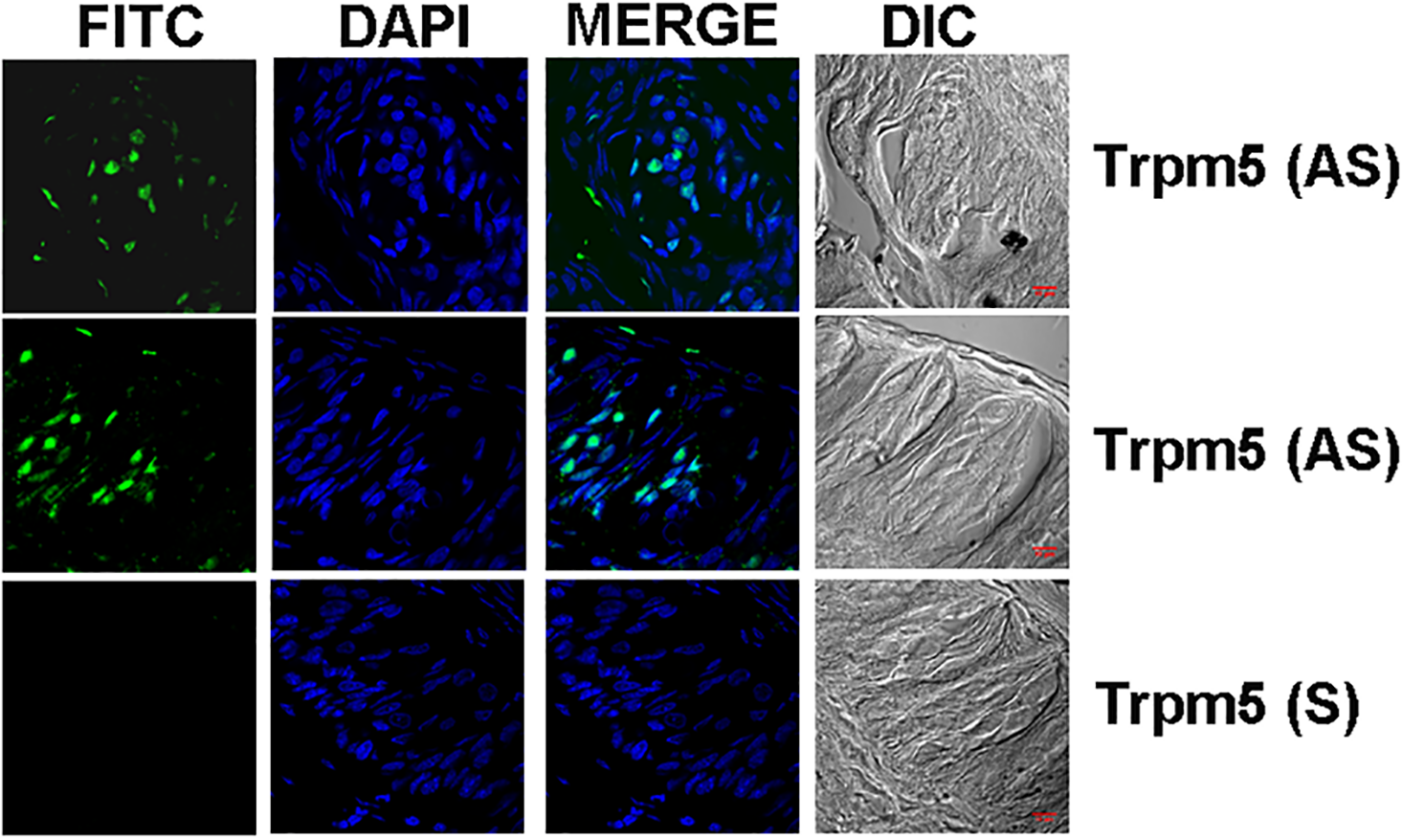
*In situ* hybridization of Trpm5 digoxigenin labeled anti-sense RNA probe in rat CV taste papillae sections. Shows low magnification images of rat CV papilla sections labelled with Alexa Fluor® 488 (FITC), DAPI, the merged images of FITC and DAPI, and the DIC image. The anti-sense (AS) Trpm5 riboprobe labeled a subset of rat CV taste bud cells (AS). No significant fluorescence was observed when the sense (S) probe was used. Bar = 10 μm.

In an additional rat CV taste papilla section, the AS Chrna7 riboprobe revealed only a subset of TRCs that express mRNA for α7 nAChR (Fig 3). To further validate our ISH technique, we show that the AS Trpm5 riboprobe also revealed only a subset of TRCs that express the mRNA for Trpm5 (Fig 4). Additional high magnification images of AS Trpm5 riboprobe binding to a subset of CV TRCs is shown in S2 Fig. These results show that in both rat and mouse CV and FF taste buds nAChR subunit mRNAs are expressed in a subset of TRCs.

Because amplification of the signal was required to visualize the low expression levels of nAChR mRNAs, we were not able to co-localize multiple nAChR subunit mRNAs in individual taste cells using ISH technique.

### IHC studies in mouse CV and FF TRCs

**Fig 5:**
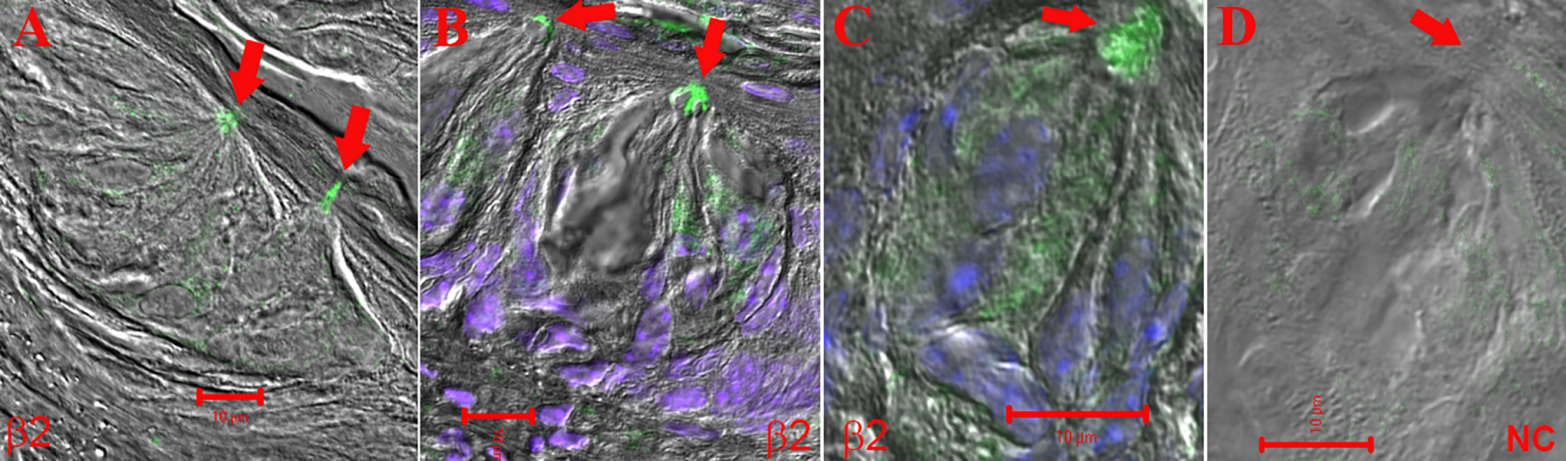
Immunostaining of β2 nAChR in WT mouse CV taste bud cells. The images are merged images of DIC, DAPI, and secondary antibody fluorescence (Alexa Fluor® 488). **(A)** A low magnification image of mouse CV taste papilla section that shows preferential binding of the nAChR β2 antibody to the apical pole of CV taste bud cells (red arrows). **(B)** and **(C)** High resolution images of the CV taste bud cells showing preferential binding of the antibody to the apical pole of the TRCs. Some binding was also observed in the intracellular/basal compartment of TRCs. **(D)** No antibody binding was observed when the primary antibody step was omitted (NC). Horizontal bars = 10 μm.

In representative CV taste papilla sections from WT mice, the β2 antibody demonstrated a preferential binding to the apical pole of a subset of CV TRCs. However, some binding was also observed in the intracellular/basolateral region of some TRCs (Fig 5A–5C; arrows). Fig 5A shows a low magnification image and Fig 5B and 5C show higher magnification images in representative sections of the CV taste papillae. The images are overlay of transmitted image, DAPI image, and FITC labelled secondary antibody. Additional high magnification images of β2 nAChR antibody binding to mouse CV papillae sections are shown in S3 Fig in panels P1 and P2. No significant antibody binding was observed in the apical pole of CV taste bud cells when the primary antibody step was omitted (Fig 5D; negative control; NC). These results suggest that the β2 nAChR subunit is most likely part of a putative apical bitter taste receptor(s) in a subset of TRCs. Since β2 nAChR antibody binding to mouse CV papillae sections was observed mainly in the apical pole of the cells, the number of β2 subunit positive cells could not be quantitated.

**Fig 6:**
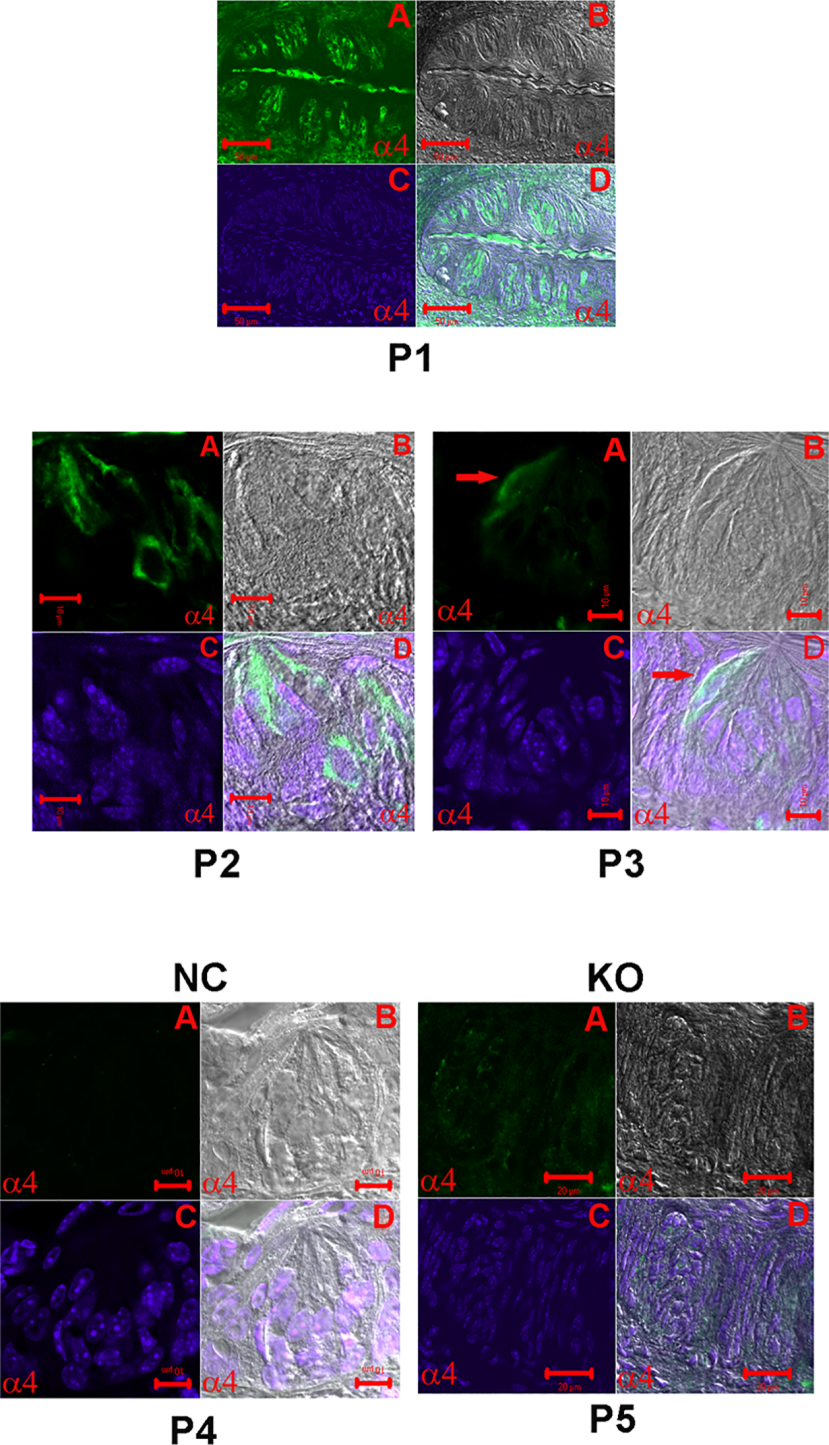
Immunostaining of α4 nAChR in WT mouse CV and FF taste bud cells. **(A)** Secondary antibody fluorescence (Alexa Fluor® 488; FITC), **(B)** DIC image, **(C)** DAPI, and **(D)** Merged images of DIC, DAPI and FITC. Panel **(P1)** shows a low magnification image of a WT mouse CV section. Only a subset of CV taste bud cells demonstrated specific binding to nAChR α4 antibody. Panels **(P2)** and **(P3)** show high magnification images of a WT mouse FF taste papillae section. Again, only a subset of FF taste bud cells showed binding to nAChR α4 antibody mainly in the basolateral/intracellular region of TRCs. Panels **(P4)** and **(P5)** show that no antibody binding was observed when the primary antibody step was omitted (NC) or in tissue sections from α4 KO mouse (KO), respectively. Horizontal bars = 10 μm.

Fig 6 (panel P1) shows a low magnification image of a CV papilla section. The image shows multiple taste buds in which α4 nAChR antibody binding was observed in a subset of TRCs within the taste buds. The higher magnification images of representative CV sections shown in Fig 6 (panels P2 and P3) demonstrate that in a subset of CV TRCs the α4 nAChR antibody binding was mainly observed in the intracellular/basolateral region of TRCs. No significant labeling was observed in TRCs when the α4 antibody step was omitted (Fig 6; panel P4; negative control; NC) or in CV sections from α4 KO mice (Fig 6; panel P5; KO). In CV sections, total number of cells were counted as DAPI labelled nuclei. In CV sections in which α4 nAChR antibody binding was investigated 31.9% TRCs were positive for α4 nAChR (Table 2).

**Table 2:** Fraction of CV TRCs labelled with nAChR antibodies.

*Total cell Number	Antibody	Positive cells	Percent
119	α4	38	31.9
157	α3	24	15.3
73	α7	13	17.8
78	β4	10	12.8

*Total number of cells were counted as cell nuclei stained with DAPI in each slide

**Fig 7:**
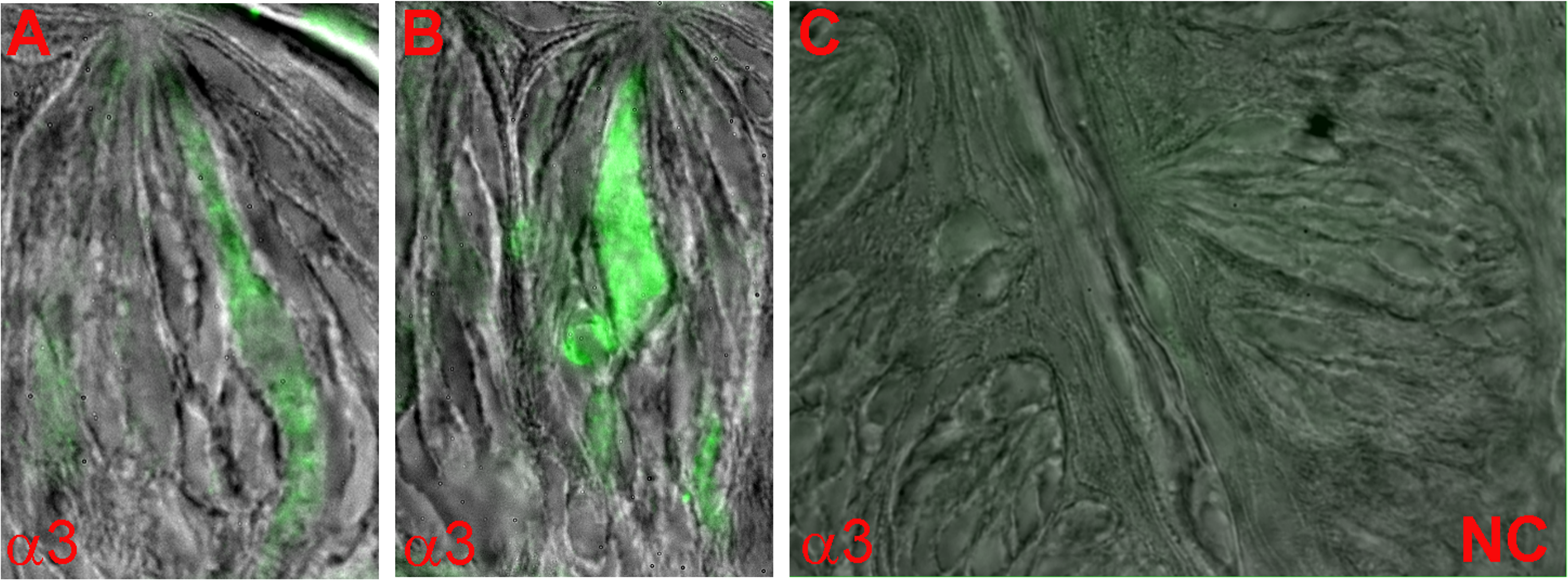
Immunostaining of α3 nAChR in WT mouse CV taste bud cells. The images are overlay of DIC image and secondary antibody fluorescence (Alexa Fluor® 488; FITC). Only a subset of CV taste bud cells showed the binding of nAChR α3 antibody (**A** and **B**). No antibody binding was observed when the primary antibody step was omitted (**C**; NC).

As shown in Fig 7A and 7B, in representative CV papilla sections, α3 nAChR antibody also showed binding to a subset of TRCs. No significant labeling was observed in TRCs when the α3 antibody step was omitted (Fig 7C; negative control; NC). In CV sections in which α3 nAChR antibody binding was investigated 15.3% TRCs were positive for α3 nAChR (Table 2).

**Fig 8:**
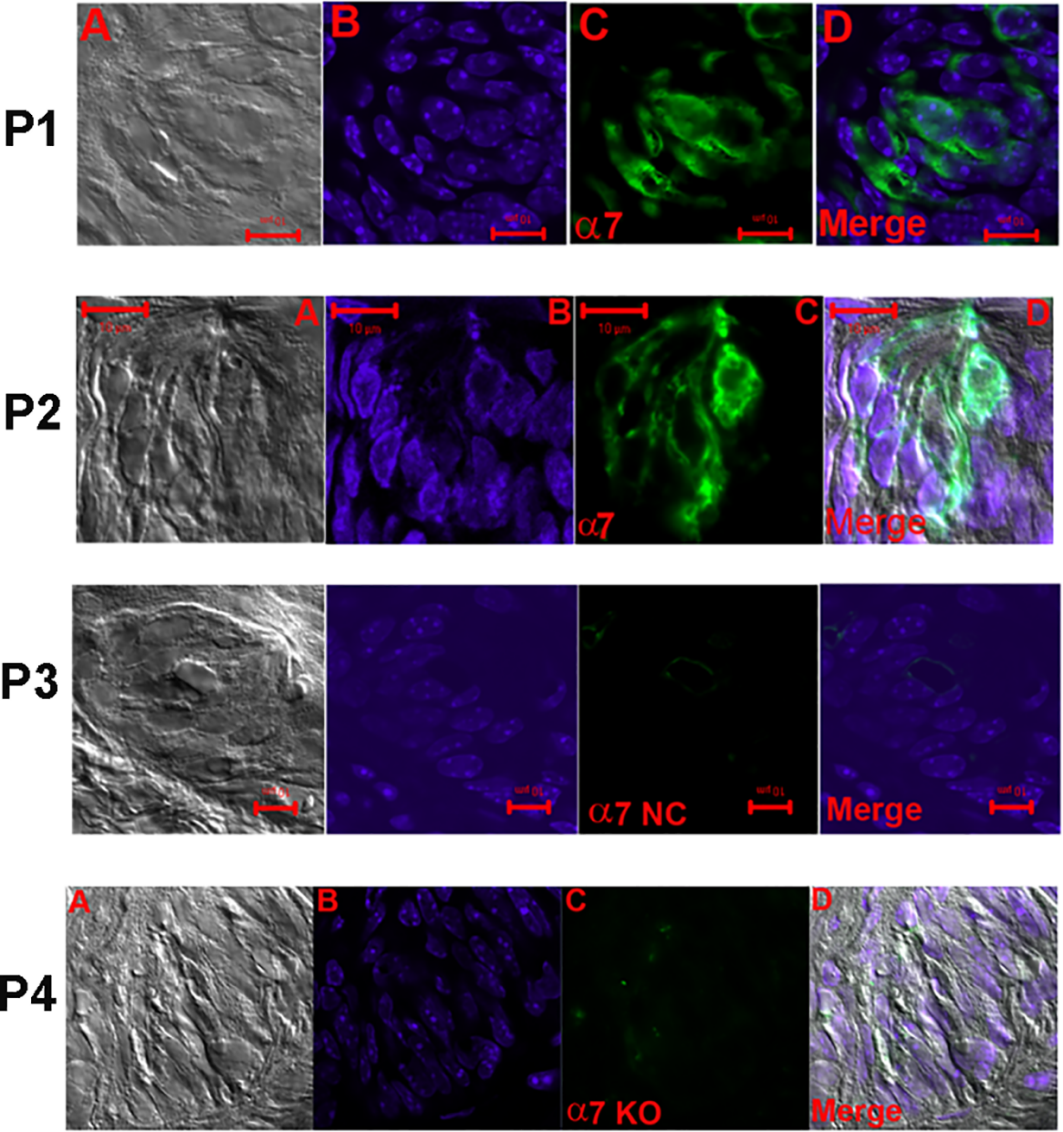
Immunostaining of α7 nAChR in WT mouse FF taste bud cells. **(A)** DIC image **(B)** DAPI; **(C)** secondary antibody fluorescence (Alexa Fluor® 488; FITC) and **(D)** Merged image of DAPI and FITC. In panels **(P1)** and **(P2)** only a subset of TRCs within the FF taste buds showed binding to the nAChR α7 antibody. In panel **(P2)** nAChR α7 antibody binding was also observed in the apical pole of TRCs. In panels **(P3)** and **(P4)** no antibody binding was observed when the primary antibody step was omitted (NC) or in tissue sections from α7 KO mouse (KO), respectively. Horizontal bars = 10 μm.

In Fig 8, panels P1 and P2 show high magnification images of representative CV sections. The images show that in CV taste buds the α7 nAChR antibody binds to the apical pole of a subset of TRCs (Fig 8; panel P2). The α7 nAChR antibody binding was also observed in the intracellular/basolateral region in a subset of TRCs. No significant labeling was observed in TRCs when the α7 antibody step was omitted (Fig 8; panel P3; NC) or in CV sections from α7 KO mice (Fig 8; panel P4; KO). In CV sections in which α7 nAChR antibody binding was investigated 17.8% TRCs were positive for α7 nAChR (Table 2).

### Alpha3, α4, α7, and β4 nAChR antibody binding in CV sections obtained from Trpm5-GFP transgenic mice

**Fig 9:**
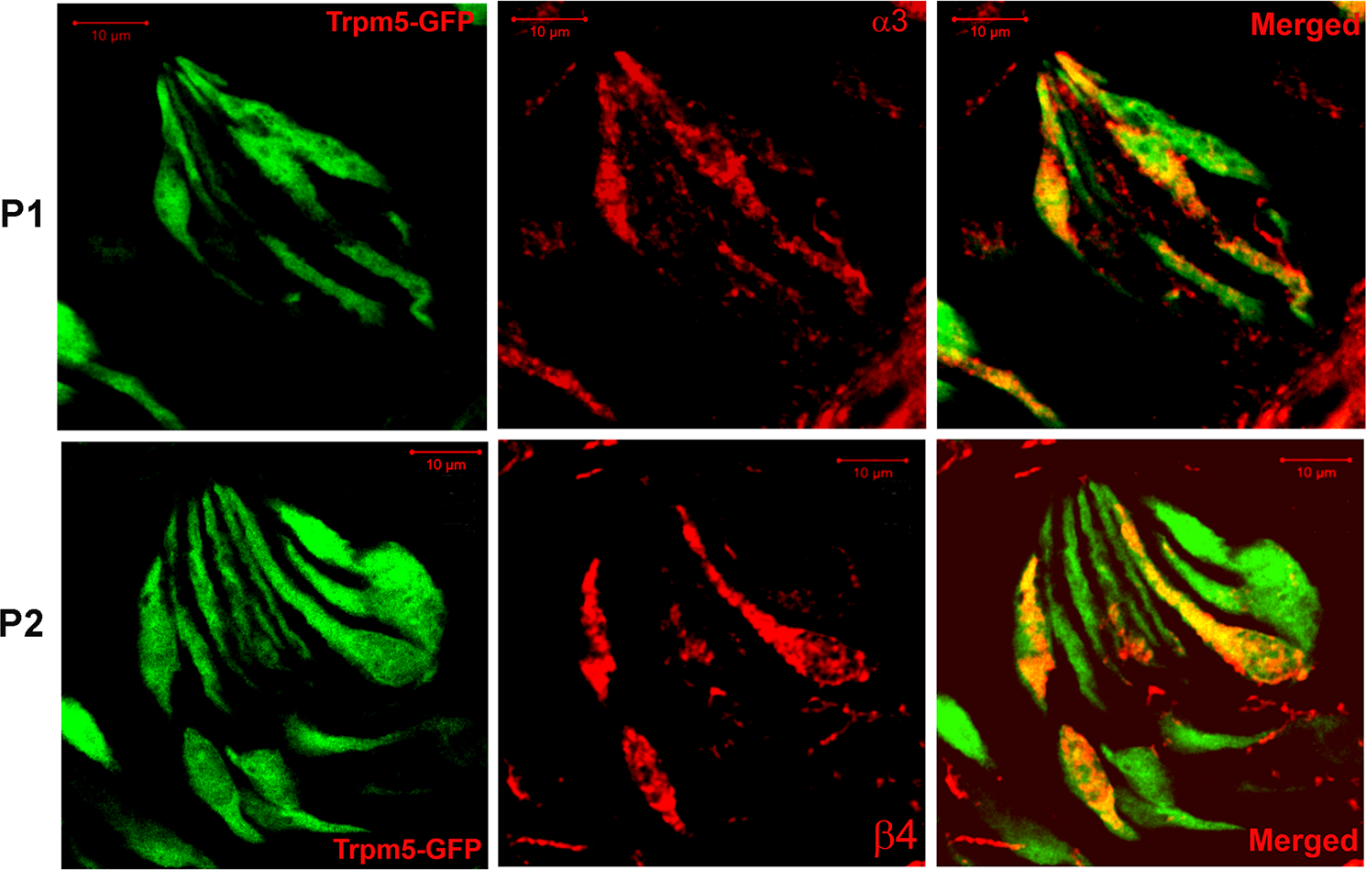
Immunostaining of α3 nAChR and β4 nAChR in CV taste bud cells from Trpm5-GFP transgenic mice. Panel **(P1)** shows images of Trpm5-GFP cells (green); nAChR α3 binding (using Cyamine 5 Amplification Reagent and secondary donkey//rabbit antibody), and the merged images of Trpm5-GFP and the 590 red-fluorescent dye. A subset of Trpm5-GFP cells demonstrate the binding of nAChR α3 antibody. Panel **(P2)** shows images of Trpm5-GFP cells (green); nAChR β4 binding (using Cyamine 5 Amplification Reagent and secondary donkey/goat antibody), and the merged images of Trpm5-GFP and the 590 red-fluorescent dye. A subset of Trpm5-GFP cells demonstrate the binding of nAChR β4 antibody. Bar = 10 μm.

**Fig 10:**
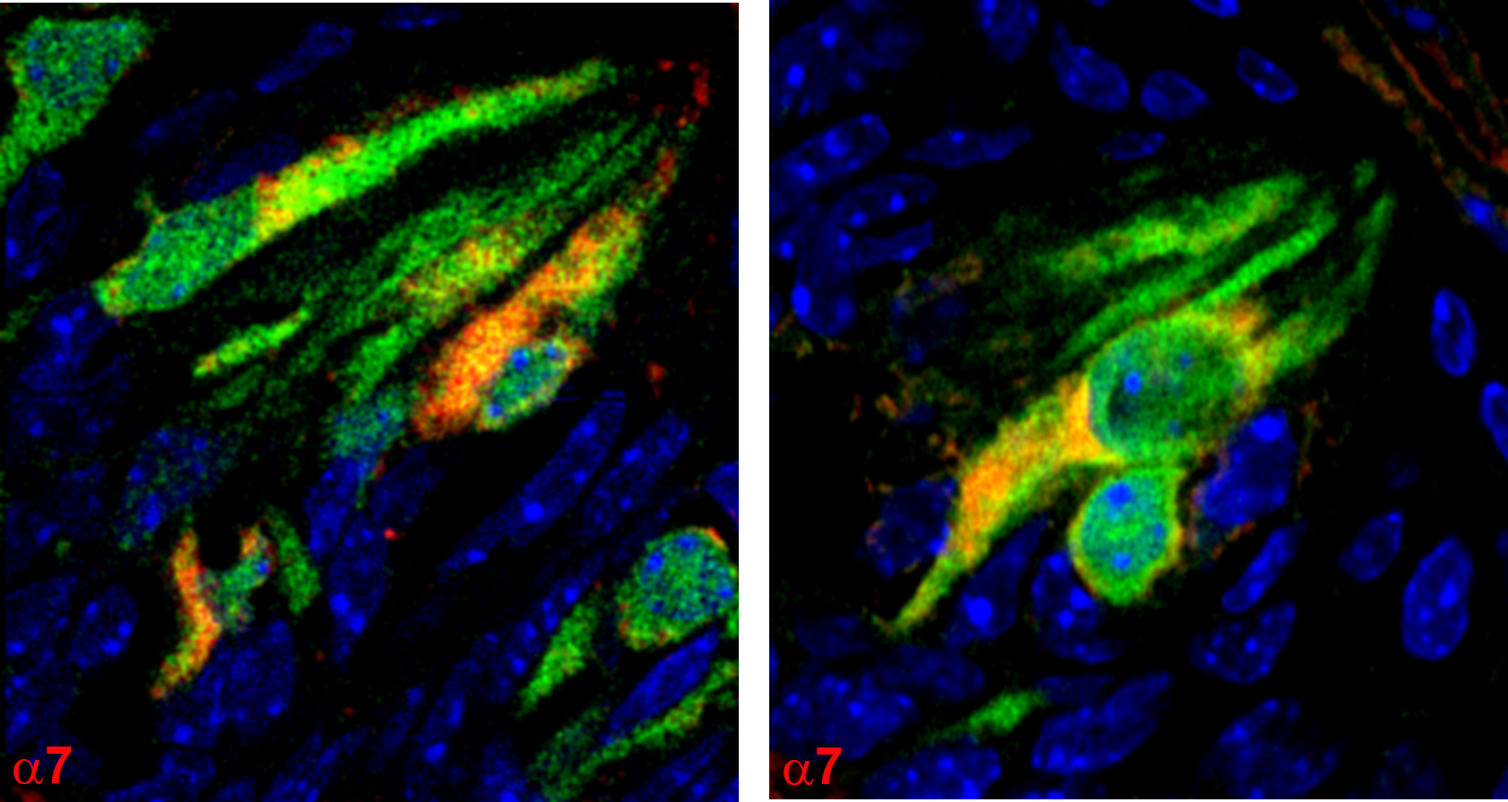
Immunostaining of α7 nAChR in CV taste bud cells from Trpm5-GFP transgenic mice. Shows merged images of Trpm5-GFP cells (green); nAChR α7 binding (using Cyamine 5 Amplification Reagent and secondary donkey//rabbit antibody) and DAPI (blue). A subset of Trpm5-GFP cells demonstrate the binding of nAChR α7 antibody.

**Fig 11:**
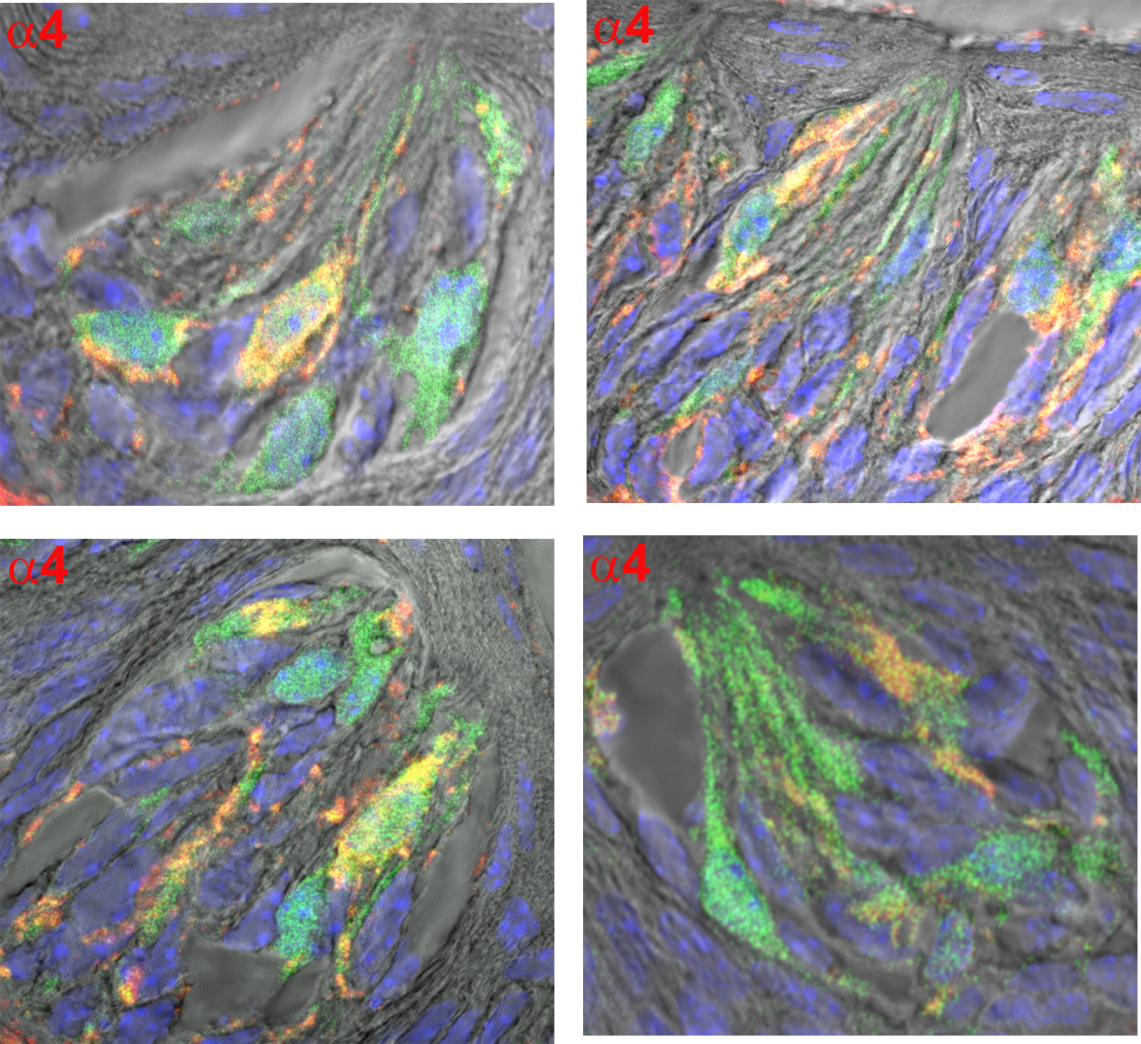
Immunostaining of α4 nAChR in CV taste bud cells from Trpm5-GFP transgenic mice. Shows merged images of Trpm5-GFP cells (green); nAChR α4 binding (using Cyamine 5 Amplification Reagent and secondary donkey/goat antibody), DAPI (blue), and the DIC image. A subset of Trpm5-GFP cells demonstrate the binding of nAChR α4 antibody.

**Fig 12:**
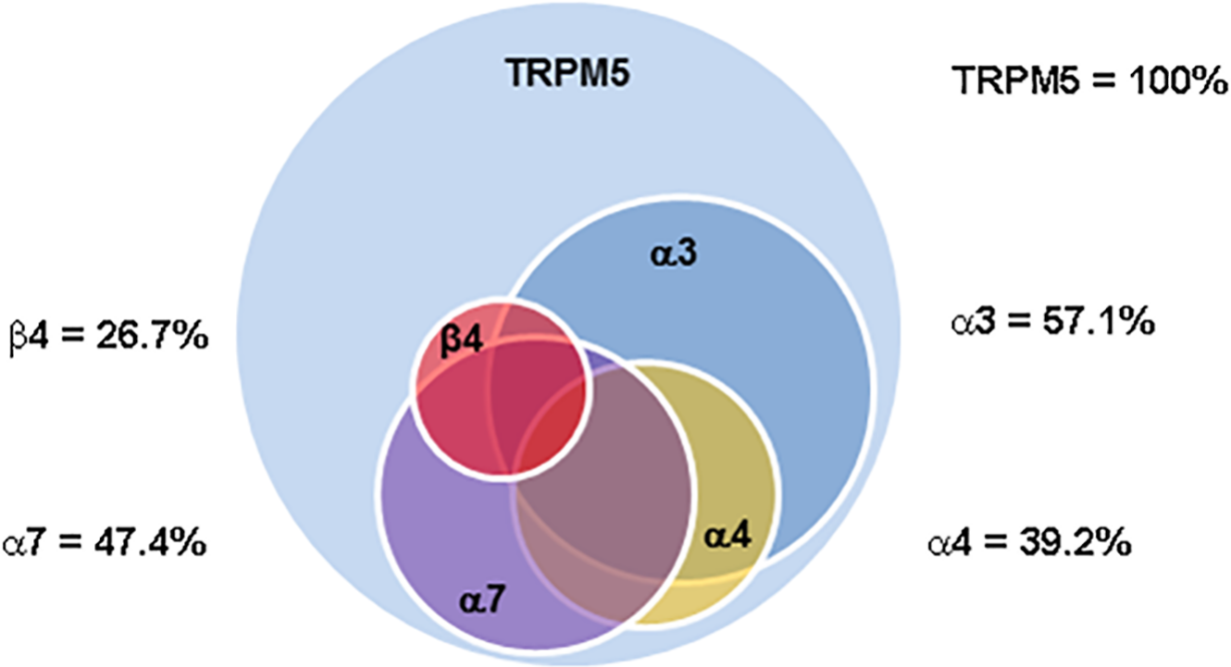
Relative distribution of nAChRs in TRPM5-GFP CV taste bud cells. Shows the percent distribution of nAChRs in TRPM5-GFP cells in various CV taste papillae sections as a Venn diagram.

We next tested if nAChR subunits are localized in Trpm5-positive TRCs. Accordingly, α3, α4, α7, and β4 nAChR antibody binding was investigated in CV sections from Trpm5-GFP transgenic mice. In CV sections from Trpm5-transgenic mice, out of 479 TRCs counted as nuclei stained with DAPI, 179 (36.3%) TRCs were GFP-positive. As shown in Fig 9, α3 (panel P1) and β4 (panel P2) nAChR antibody binding was observed in a subset of CV TRCs. In addition, the α3 and β4 nAChR antibody binding was observed in a subset of Trpm5-GFP TRCs. As shown in Figs 10 and 11, α7 and α4 nAChR antibody binding was also observed in a subset of Trpm5-GFP TRCs. In each CV section examined the number of GFP (green) and nAChR antibody stained cells (red) were counted. In CV sections in which α3 nAChR antibody binding was investigated in Trpm5-GFP cells, 57.1% TRCs were positive for α3 nAChR (Fig 12). In CV sections in which β4 nAChR antibody binding was investigated in Trpm5-GFP cells, 26.7% TRCs were positive for β4 nAChR (Fig 12). In CV sections in which α4 and α7 nAChR antibody binding was investigated in Trpm5-GFP cells 39.2% and 47.4% TRCs were positive for α4 and α7 nAChRs, respectively (Fig 12).

### Alpha3 nAChR antibody binding in intestinal enteroendocrine cells derived from Trpm5-GFP transgenic mice

**Fig 13:**
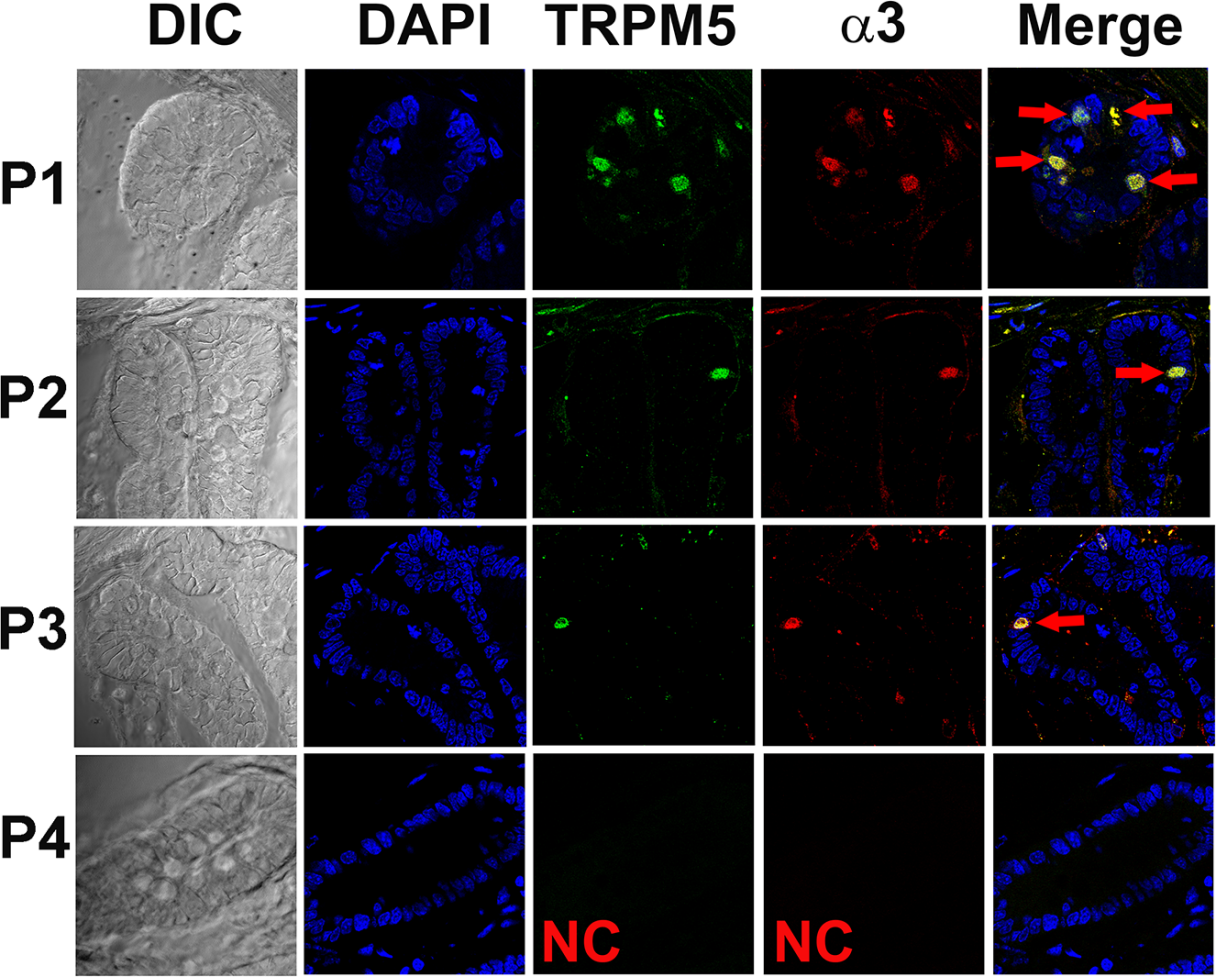
Immunostaining of α3 nAChR in enteroendocrine cells in the Trpm5-GFP mouse gut. Shows DIC image, DAPI, Trpm5-GFP, α3 nAChR antibody binding (secondary Donkey/Rabbit-590 antibody), and the merged images of DAPI, GFP and 590 red-fluorescent dye. In each of the 8 slides from 3 different gut sections examined 1 to 4 enteroendocrine cells were positive for α3 nAChR in the crypts (**P1, P2, and P3;** red). The α3 nAChR-positive cells were also positive for Trpm5-GFP (green). Negative control (NC) without primary antibody **(P4)**.

To further confirm that nAChRs are co-expressed with Trpm5, we investigated the binding of α3 nAChR antibody in Trpm5-GFP transgenic mouse intestinal enteroendocrine cells. As shown in Fig 13 (panels P1-P3), α3 nAChR antibody was observed in a subset of enteroendocrine cells in the crypt that were also positive for Trpm5. These results suggest that in the crypt, cells that express Trpm5 also express nAChR subunits.

### Effect of chronic oral exposure to Nic or ETOH on nAChR mRNA expression in WT mouse CV taste bud cells

**Fig 14:**
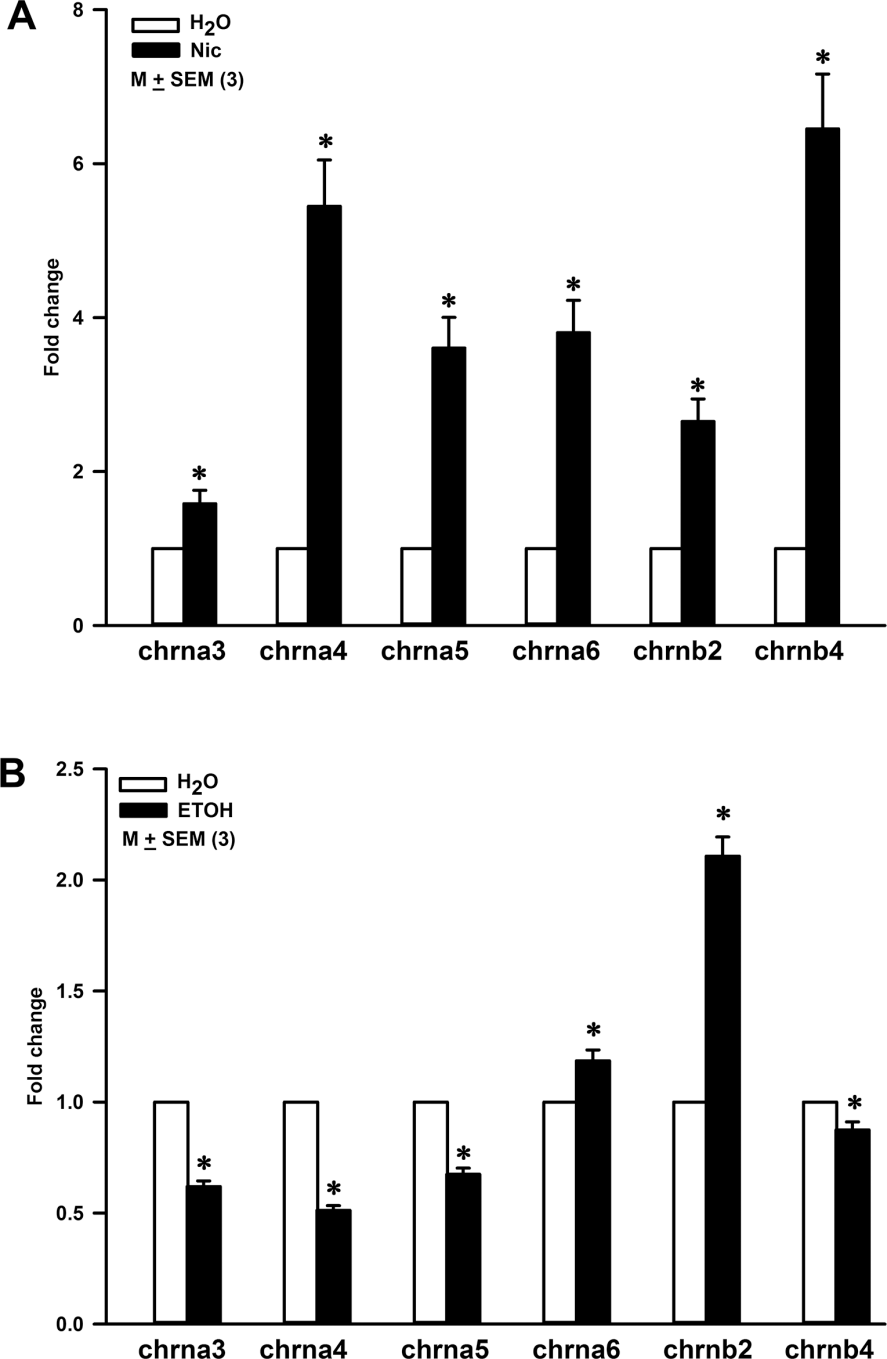
Effect of nicotine (Nic) and ethanol (ETOH) exposure on the nAChR mRNA expression levels in CV taste bud cells in WT mice. **(A)** Relative to control WT mice (H_2_O), in WT mice exposed to Nic (100 μg/ml) for 3 weeks, the p values for fold change in chrna3, chrna4, chrna5, chrna6, chrnb2 and chrnb4 mRNAs were 0.0292, 0.0018, 0.0028, 0.0026, 0.0049, and 0.0016, respectively. **(B)** Relative to control WT mice (H_2_O) mice, in WT mice exposed to ETOH (5%) for 3 weeks p values for chrna3, chrna4, chrna5, chrna6, chrnb2 and chrnb4 were 0.0001, 0.0001, 0.0003, 0.0194, 0.0002, and 0.0253, respectively. The values are mean ± SEM of triplicate runs.

STC-1 cell line is an established cell line of enteroendocrine cells of mouse small intestine. We have recently shown that exposing STC-1 cells to Nic acutely (24 h) or chronically (4 days) induced a differential increase in the expression of nAChR subunit mRNA and protein in a dose- and time-dependent fashion [8]. Here, we tested, if chronic Nic or ETOH exposure also alters nAChR mRNA expression in CV taste bud cells. Q-RT-PCR was performed on CV taste bud cells obtained from WT mice maintained on H_2_O, Nic (100 μg/ml) or ETOH (5%) for 3 weeks. Relative to mice maintained on H_2_O, mice treated with Nic showed a differential increase in mRNA expression levels for α3, α4, α5, α6, β2, and β4 nAChR subunits (Fig 14A). The largest increase was observed for α4 and β4 nAChR subunit mRNAs.

In our qRT-PCR studies, we also quantified the mRNA expression of α7 nAChR in CV taste buds. However, the fold change in the mRNA expression level of α7 nAChR in CV taste buds from Nic treated WT mice (Ct value = 38.9) was difficult to calculate because of the undetectable levels of α7 nAChR mRNA (undetectable Ct value) in control WT mice.

**Fig 15:**
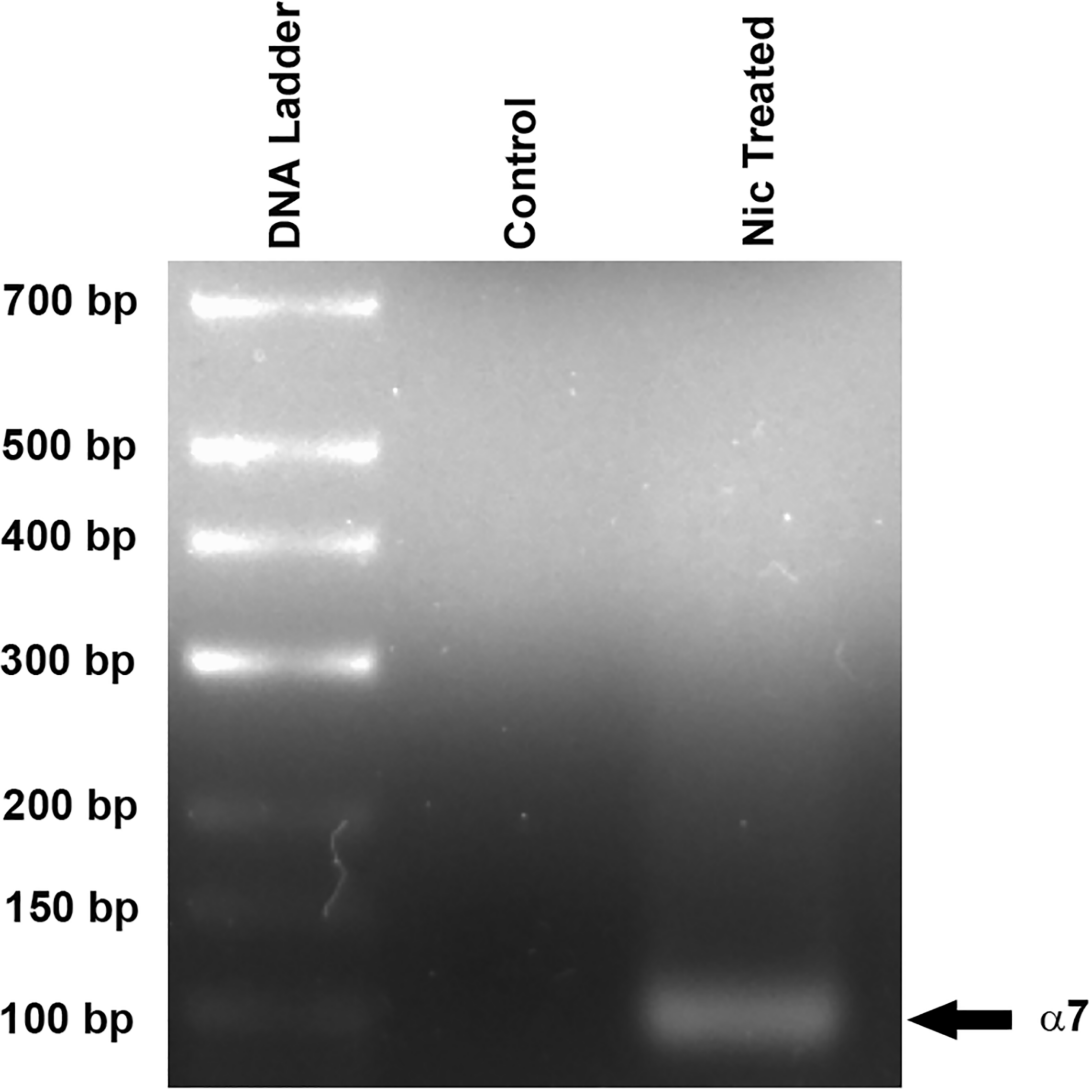
Detection of qRT-PCR α7 product by gel electrophoresis. Following qRT-PCR, the final qRT-PCR product was loaded onto 2% agarose gel to detect the presence of the amplified product.

Following qRT-PCR, the final qRT-PCR product was loaded onto 2% agarose gel to detect the present of the amplified product. No α7 nAChR DNA band was detected in CV taste buds from the control mice. However, a clear 88 bp band of α7 nAChR was observed from Nic treated WT mice (Fig 15). These data indicate that Nic treatment induces upregulation mRNA expression of α7 nAChR compared to undetectable level in control mice.

Relative to WT mice (Fig 14B) maintained on H_2_O, mice given 5% ETOH for 3 weeks demonstrated a significant decrease in the mRNA levels of α3, α4, α5, and β4 nAChR subunits. In contrast, a significant increase was observed in the mRNA levels of α6 and β2 nAChR subunits. These results suggest that chronic exposure to Nic or ETOH produces a differential effect on the mRNA levels of nAChR subunits. We hypothesize that differential changes in the nAChR expression after chronic Nic or ETOH exposure may be related to the long term adaptation to the bitter taste of Nic and ETOH.

## Discussion

Nic and ETOH evoke behavioral and CT nerve responses in rats, WT mice, and Trpm5 KO mice. Modulating the activity of nAChRs alters the CT response and the behavioral response to Nic and ETOH in rats, WT mice, and Trpm5 KO mice [2, 3]. These functional studies strongly suggested that nAChRs are expressed in a subset of FF TRCs. Consistent with this, in a cDNA library from rat FF and CV taste buds, RT-PCR experiments demonstrated the presence of mRNAs of α3, α4, β2, and β4 nAChR subunits [2]. These results provided the first preliminary evidence for the expression of nAChRs in TRCs. This paper is an extension of our previous studies. Here, we used ISH, IHC, and qRT-PCR techniques to investigate in detail the expression of nAChR subunit mRNAs and proteins in a subset of CV and FF TRCs.

### nAChR mRNA expression in TRCs

Neuronal nAChRs function as fast ionotropic cationic nicotinic receptor channels and assemble to form functional homopentameric or heteropentameric complexes. Eight α-like subunits (α2, α3, α4, α5, α6, α7, α9, and α10) and three non-α subunits (β2, β3, and β4) have been identified in neuronal tissues [24, 25]. Before applying the ISH technique on TRCs, it was fist validated in the brain tissue. In the brain tissue, nAChR mRNA expression levels are high and, therefore, no amplification of the signal was needed for detecting them in neurons and for observing co-localization of nAChR subunits in individual neurons. Using the ISH method, we detected neurons in the brain that expressed β2, α4, and α7 nAChRs mRNAs (S1 Fig). Neurons that expressed α4 nAChR mRNA also expressed the β2 nAChR mRNA and neurons that expressed α7 nAChR mRNA also expressed the β2 nAChR mRNA (S1 Fig). Greater than 90% of the high-affinity nicotinic receptors in the brain are composed of α4β2* nAChR subunits [26]. Rat basal forebrain cholinergic neurons co-express α7 and β2 nAChR subunits. These subunits combine to form a unique heteromeric α7β2 nAChR. The heteromeric α7β2 nAChR displays distinct biophysical properties from those of the homomeric α7 nAChR in ventral tegmental area neurons [27].

Due to the low expression of nAChRs in the taste tissue, we were only able to detect the ISH signal in FF and CV taste bud cells using amplification. This precluded us from being able to co-localize the mRNA for multiple nAChR subunit in individual taste cells in Trpm5-GFP mice.

Using signal amplification the mRNA for β2, β4 and α7 were detected in a subset of CV and FF TRCs (Figs 1–3). The ISH technique in TRCs was further validated by the demonstration that mRNA for Trpm5 ion channel is also expressed in a subset of CV TRCs (Fig 4 and S2 Fig). Trpm5 ion channel is involved in sweet, bitter and umami taste transduction [28]. The IHC results indicate that like the Trpm5 ion channel, nAChR mRNAs are also expressed only in a subset of CV and FF TRCs derived from mouse or rat CV and FF taste papillae.

### nAChR protein expression in TRCs

Consistent with the expression of mRNAs in a subset of CV and FF TRCs, our IHC studies demonstrate that only a subset of CV and FF TRCs bind to specific antibodies to α3, α4, α7, β2, and β4 nAChRs (Figs 5–12 and S3 Fig). Of particular significance is the observation that α7- and β2-antibodies preferentially labelled the apical pole of a subset of CV and FF taste bud cells (Figs 5 and 8). This suggests, but does not prove, that a putative nAChR composed of the α7 subunit and the β2 subunit may function as the bitter taste receptor for Nic and/or ETOH in the apical membrane of a subset of CV and FF TRCs. As stated above, α7 and β2 nAChR subunits form a novel heteromeric α7β2 nAChR subtype. However, α and β subunits can combine to form simple or complex receptors with multiple subunits [24, 25]. The subunit composition of nAChRs involved in the apical nAChR receptor(s) for Nic and ETOH remain to be elucidated.

Although, the vast majority of the antibodies against nAChR subunits are considered to lack specificity [29, 30], in this study nAChR expression in TRCs was confirmed by qRT-PCR, ISH and IHC techniques. Strong supporting evidence for the presence of nAChRs in TRCs is provided by the effect of nAChR agonists and antagonists on the neural and behavioral responses in WT mice, Trpm5 KO mice and rats [2, 3] and by our recent studies in STC-1 cells [8].

Using specific α3-, α4-, α7- or β4-antibodies in CV taste buds derived from Trpm5-GFP mice showed that α3, α4, α7, and β4 nAChR subunits are expressed in a subset of Trpm5 positive TRCs (Figs 9–12). These results are supported by our observations that in intestinal crypt cells derived from Trpm5-GFP transgenic mice the α3-nAChR subunit specific antibody also labelled Trpm5-positive cells (Fig 13). In epithelial cells of intrapulmonary airways, nAChRs have been shown to be co-expressed in cell that express the bitter taste receptors and downstream signaling effectors [10]. These studies tend to suggest that the detection of the bitter taste of Nic and ETOH by Trpm5-dependent and Trpm5-independent mechanism are most likely present in the same Trpm5-positive cells.

In our previous studies, treating Trpm5 KO mice with Mec, inhibited CT response to Nic and reduced the behavioral responses of aversion to Nic [2]. These results suggest that even though nAChRs are expressed in Trpm5 positive cells, the nAChRs function independently of Trpm5. Patch clamp studies were performed on HEK-293 cells overexpressing Trpm5 ion channel [31]. In these studies, at -50 mV, Nic inhibited TRPM5 currents with an effective inhibitory concentration of ~1.3 mM. These results suggest that at high concentrations, the bitter taste of Nic is transduced by Trpm5-independent mechanism(s). In brief-access tests, the aversion to Nic was decreased in the presence of Mec [2]. These results suggest that at high Nic concentrations the bitter taste of Nic is transduced by nAChR-dependent but Trpm5-independent mechanism(s).

In our previous study [3], we monitored the magnitude of the tonic CT responses to Nic in WT and Trpm5 KO mice in the absence and presence of Mec. From these data, it was estimated that at Nic concentrations < 5 mM, 50% of the total tonic CT response was contributed by the interaction of Nic with the nAChR-dependent pathway. The other 50% of the tonic CT response was due to the interactions of Nic with the T2r-Trpm5-dependent pathway. At Nic concentrations >5 mM, the T2r-Trpm5-dependent component of the CT response achieved a maximum value and the nAChR-dependent component of the CT response predominated. At 10 mM and 20 mM Nic, the contribution of the T2r-Trpm5-dependent pathway to the CT response decreased to 41% and 35%, respectively. At Nic concentrations greater than 20 mM, the T2r-Trpm5-dependent CT response component further decreased to 30% [3]. While nAChRs play a significant role in detecting the bitter taste of Nic and ETOH, the olfactory responses to Nic are independent of nAChRs [32].

### Oral exposure to Nic and ETOH alters nAChR mRNA profile in CV taste bud cells

We have recently [8] shown that exposing STC-1 cells to 0.25, 0.50, and 1.0 μM Nic acutely (24 h) or chronically (4 days) induced an increase in the mRNA expression of α4, α5, and α6. The changes in α4, α5, and α6 mRNA varied with both Nic concentration and the exposure time. In WT mice exposed to Nic for 3 weeks, we also observed significant increase in mRNA expression of α4, α5, α6, α7, and β4 in CV taste bud cells (Figs 14A and 15). These changes were accompanied by smaller changes in α3 and β2 mRNA. These results indicate that chronic Nic exposure induces up-regulation of α4, α5, α6, and α7 nAChR subunits in both STC-1 cells and CV taste bud cells. While Nic induced significant increase in the expression of β2 and β4 mRNA in CV taste bud cells, chronic Nic exposure did not produce an increase in β4 mRNA in STC-1 cells. This suggests that Nic exposure produces differential effects on nAChR mRNAs in different cell types.

Consistent with our studies, chronic Nic exposure has been shown to upregulate α4 nAChR subunit in the hippocampal medial prefrontal path and on ventral tegmental area GABAergic neurons [33], two-fold increase in the β2-containing receptors and a smaller up-regulation in the α4-containing nAChRs in rat primary cultured neurons [34], and up-regulation of β2 containing nAChRs in lateral septum, caudate putamen, and nucleus accumbens [35]. This suggests that chronic Nic exposure induces up-regulation of nAChR subtypes in tissue and cell specific manner.

In contrast to Nic, chronic ETOH exposure for 3 weeks induced a decrease in the mRNA expression of α3, α4, α5, and β4 nAChR subunits. However, there was an increase in the mRNA expression of α6, and β2 nAChR subunits after chronic ETOH exposure (Fig 14B). These results suggest that Nic and ETOH regulate receptors composed of different combination of nAChR subunits in CV taste bud cells. Thus, although nAChRs are common targets at which Nic and ETOH interact in the central nervous system [24, 25] and in TRCs [2, 3, 36, 37] they most likely act on separate receptors comprising different combination of nAChR subunits. In this regard, Mec, CP-601932, sazetidine A and varenicline reduce ETOH and Nic consumption and seeking [3, 38–40] and the pleasurable effects of alcoholic beverages in patients [41]. The nAChRs also play an important role in the neurobiological effects of ethanol. There is a significant associations between excessive ethanol-drinking behavior and polymorphisms in CHRNA6, CHRNB3, and CHRNA4 [16]. It is suggested that α4β2 and α7 nAChR subtypes may not be essential for ethanol reward and consumption behavior. In contrast, α6* and/or α3* nAChR subtypes probably serve as important targets for ETOH-induced behavioral alterations [42].

In summary, using q-RT-PCR, ISH and IHC techniques we show for the first time that nAChR subunits are expressed in a subset of rat and mouse CV and FF TRCs. The nAChRs are expressed in a subset of Trpm5-positive TRCs. These results are consistent with the functional observations that agonists and antagonists of nAChRs modulate neural and behavioral responses in WT mice, Trpm5 KO mice and rats. The results further indicate Nic evokes bitter taste via two receptor-dependent mechanisms. One pathway is T2r-Trpm5-dependent and the other pathway is T2r-Trpm5-independent but is dependent upon the presence of nAChRs expressed in a subset of Trpm5-positive TRCs. In addition to Nic, the bitter taste of ETOH is also nAChR-dependent. Currently, it is not known if nAChRs co-localize in Trpm5 cells that also express the bitter taste receptors, T2rs. Our results further show that in WT mice, chronic oral exposure to Nic and ETOH induce changes in specific nAChRs in CV taste bud cells. We hypothesize that changes in specific nAChRs may be related to the taste-adaption with chronic Nic or ETOH exposure.

## Supporting information

S1 Fig *In situ* hybridization of digoxigenin labeled Chrnb2, Chrna4, and Chrna7 anti-sense (AS) RNA probes in a brain section from WT mice. Panel **(P1)** shows a transmitted image (DIC) of the brain section **(A)** and the fluorescence signal in the same brain section **(B)**. The AS riboprobe Chrnb2 labeled individual neurons in brain sections. Panels **(P2)** and **(P3)** show DIC image **(A)**, DAPI **(B)**, Alexa Fluor® 488 **(C)**, Alexa Fluor® 590 red-fluorescent dye **(D)**, and merged images of A, B, C, and D **(E)**. Panel **(P2)** shows the labeling of the AS riboprobe Chrna4 (green) and AS riboprobe Chrnb2 (red) in individual neurons in brain sections. A subset of neurons show dual labeling of the AS riboprobe Chrna4 (green) and AS riboprobe Chrnb2 (red) in individual neurons. Panel (**P3)** shows the labeling of the AS riboprobe Chrna7 (green) and AS riboprobe Chrnb2 (red) in individual neurons in brain sections. A subset of neurons show dual labeling of AS riboprobe Chrna7 (green) and AS riboprobe Chrnb2 (red) in individual neurons.
(TIF)

S2 Fig *In situ* hybridization of Trpm5 digoxigenin labeled anti-sense RNA probe in rat CV taste papillae sections. Shows high magnification merged images of rat CV papilla sections labelled with Alexa Fluor® 488 (FITC) **(A-C)** and the corresponding DIC images **(D-E)**. The AS Trpm5 riboprobe labeled a subset of rat CV taste bud cells.
(TIF)

S3 Fig Immunostaining of β2 nAChR in WT mouse CV taste bud cells. **(A)** DIC image, **(B)** secondary antibody fluorescence (Alexa Fluor® 488), **(C)** DAPI, and **(D)** merged image of DAPI and Alexa Fluor® 488. Panels **(P1)** and **(P2)** show high magnification images of the CV taste bud cells showing preferential binding of the antibody to the apical pole of the TRCs (red arrows). Some binding was also observed in the intracellular/basal compartment of TRCs. Horizontal bars = 10 μm.
(TIF)
