# Supplementary figures and images for: Nicotinic acetylcholine receptors (nAChRs) are expressed in Trpm5 positive taste receptor cells (TRCs)
Source: PLoS One. 2018 Jan 2;13(1):e0190465. doi: 10.1371/journal.pone.0190465 (PMC5749851; doi:10.1371/journal.pone.0190465)

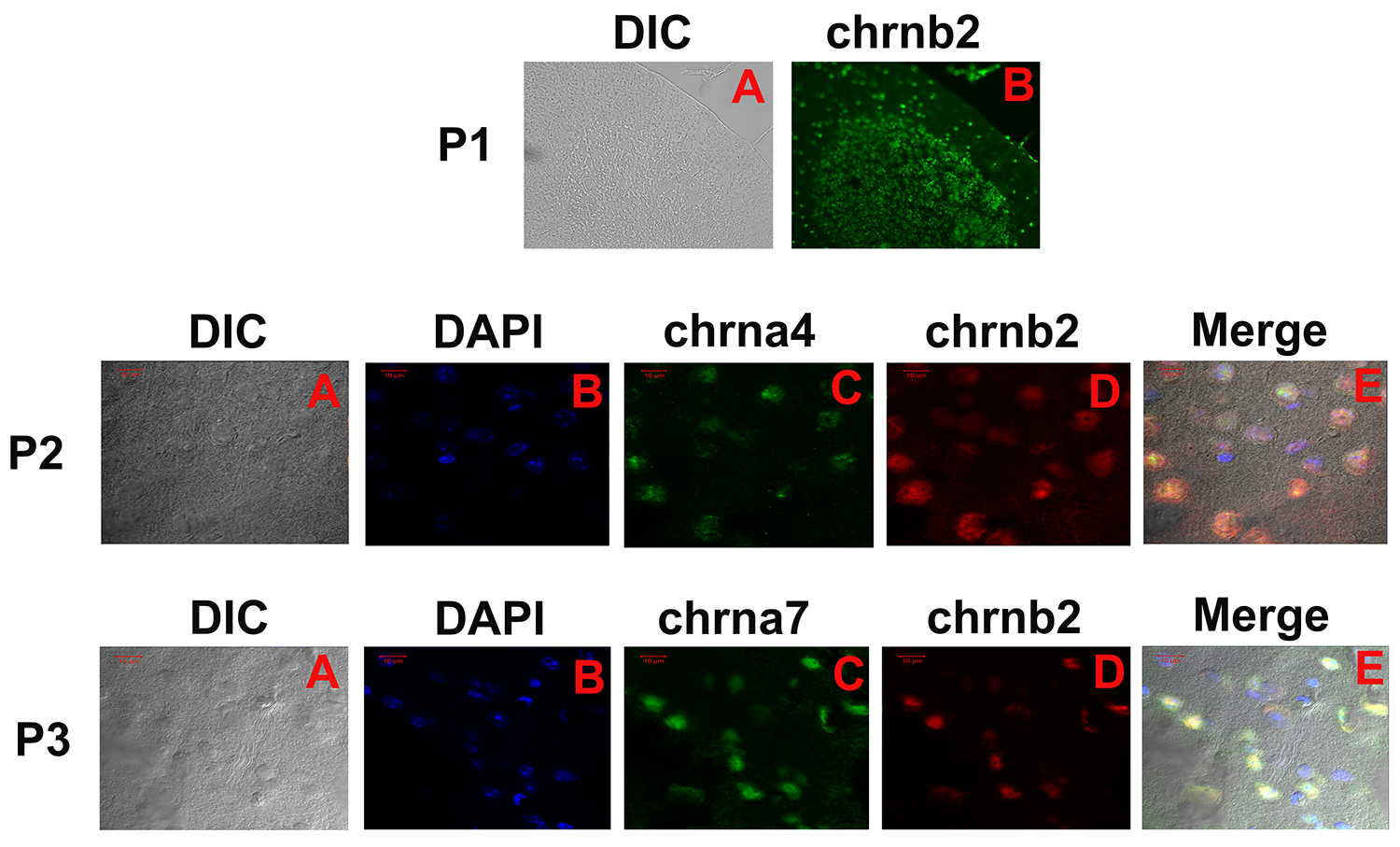

Supplement: S1 Fig — Panel (P1) shows a transmitted image (DIC) of the brain section (A) and the fluorescence signal in the same brain section (B). The AS riboprobe Chrnb2 labeled individual neurons in brain sections. Panels (P2) and (P3) show DIC image (A), DAPI (B), Alexa Fluor® 488 (C), Alexa Fluor® 590 red-fluorescent dye (D), and merged images of A, B, C, and D (E). Panel (P2) shows the labeling of the AS riboprobe Chrna4 (green) and AS riboprobe Chrnb2 (red) in individual neurons in brain sections. A subset of neurons show dual labeling of the AS riboprobe Chrna4 (green) and AS riboprobe Chrnb2 (red) in individual neurons. Panel (P3) shows the labeling of the AS riboprobe Chrna7 (green) and AS riboprobe Chrnb2 (red) in individual neurons in brain sections. A subset of neurons show dual labeling of AS riboprobe Chrna7 (green) and AS riboprobe Chrnb2 (red) in individual neurons. (TIF) [file pone.0190465.s001.tif]

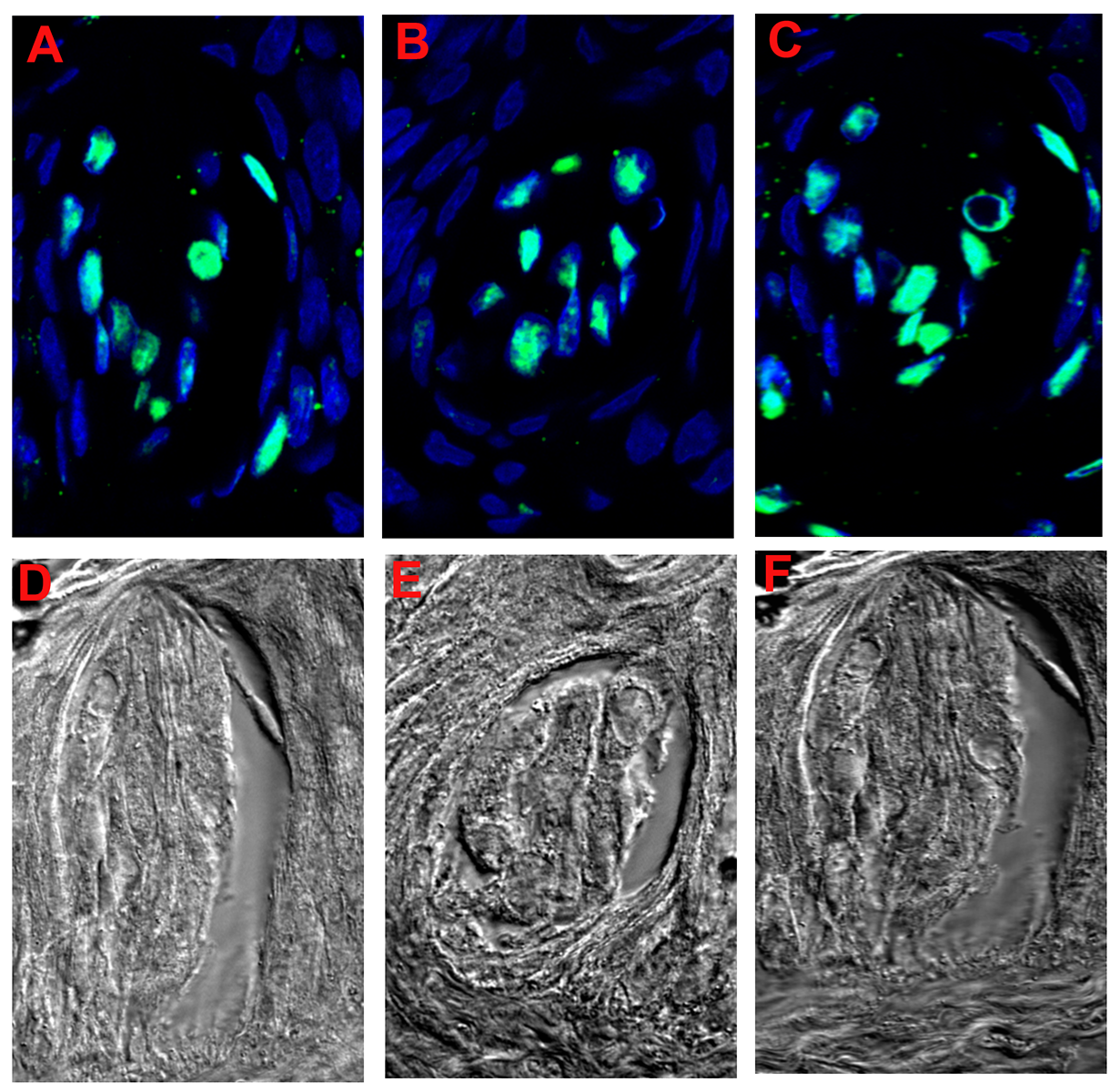

Supplement: S2 Fig — Shows high magnification merged images of rat CV papilla sections labelled with Alexa Fluor® 488 (FITC) (A-C) and the corresponding DIC images (D-E). The AS Trpm5 riboprobe labeled a subset of rat CV taste bud cells. (TIF) [file pone.0190465.s002.tif]

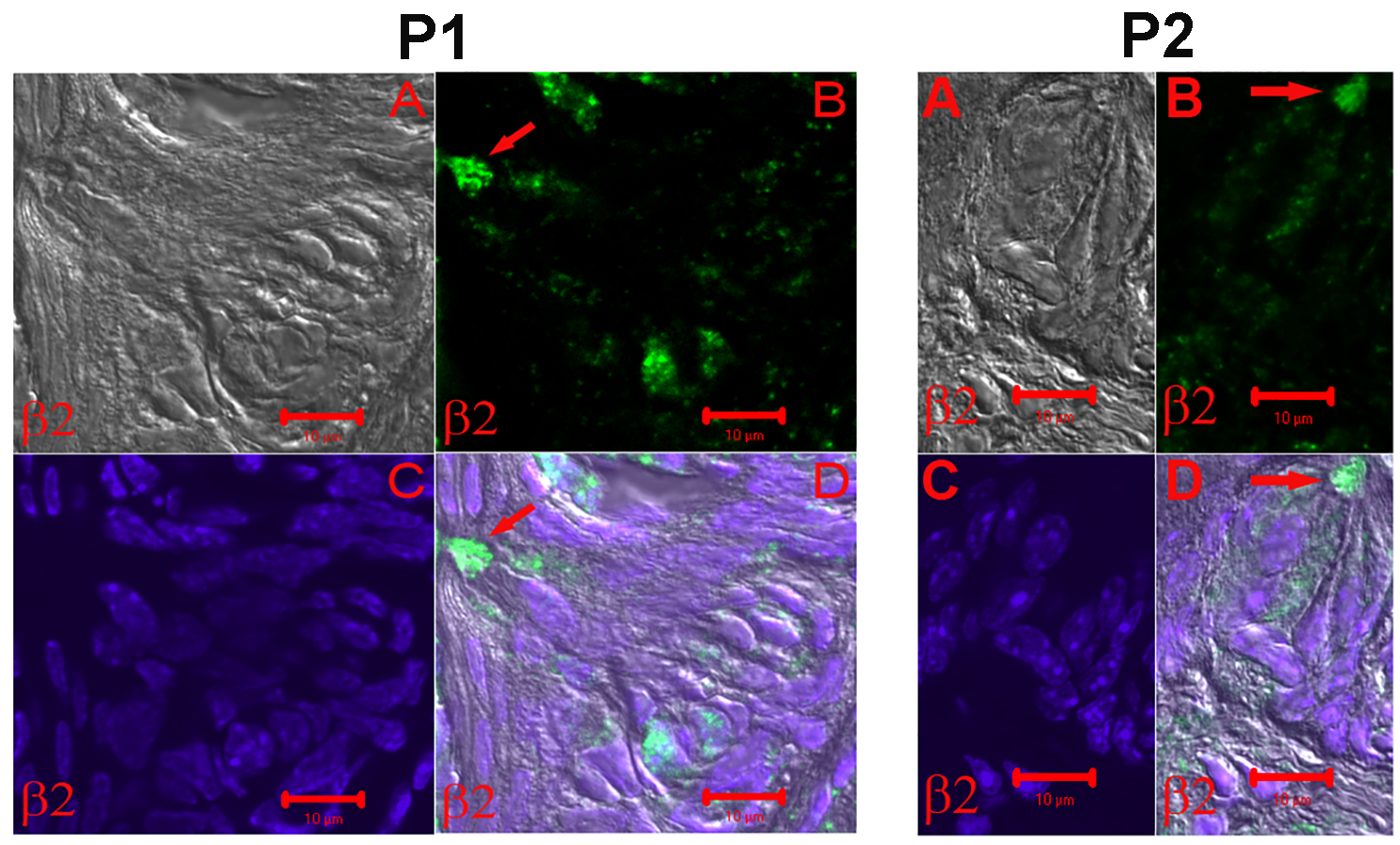

Supplement: S3 Fig — (A) DIC image, (B) secondary antibody fluorescence (Alexa Fluor® 488), (C) DAPI, and (D) merged image of DAPI and Alexa Fluor® 488. Panels (P1) and (P2) show high magnification images of the CV taste bud cells showing preferential binding of the antibody to the apical pole of the TRCs (red arrows). Some binding was also observed in the intracellular/basal compartment of TRCs. Horizontal bars = 10 μm. (TIF) [file pone.0190465.s003.tif]
